# Glia maturation factor-*γ* is required for initiation and maintenance of hematopoietic stem and progenitor cells

**DOI:** 10.1186/s13287-023-03328-1

**Published:** 2023-04-30

**Authors:** Honghu Li, Qian Luo, Shuyang Cai, Ruxiu Tie, Ye Meng, Wei Shan, Yulin Xu, Xiangjun Zeng, Pengxu Qian, He Huang

**Affiliations:** 1 Bone Marrow Transplantation Center, School of Medicine, The First Affiliated Hospital, Zhejiang University, No. 79 Qingchun Road, Hangzhou, 310012 Zhejiang People’s Republic of China; 2 Liangzhu Laboratory, Zhejiang University Medical Center, 1369 West Wenyi Road, Hangzhou, 311121 People’s Republic of China; 3 Institute of Hematology, Zhejiang University, Hangzhou, People’s Republic of China; 4 Zhejiang Province Engineering Laboratory for Stem Cell and Immunity Therapy, Hangzhou, People’s Republic of China; 5 Center of Stem Cell and Regenerative Medicine, School of Medicine, Zhejiang University, Hangzhou, 310012 People’s Republic of China; 6 Dr. Li Dak Sum & Yip Yio Chin Center for Stem Cell and Regenerative Medicine, Zhejiang University, Hangzhou, 310012 Zhejiang People’s Republic of China; 7 School of Medicine, Zhejiang University, No. 866 Yuhangtang Road, Hangzhou, People’s Republic of China

**Keywords:** Glia maturation factor-*γ*, HSPC, Blood flow, Yap, Notch, Zebrafish

## Abstract

**Background:**

In vertebrates, hematopoietic stem and progenitor cells (HSPCs) emerge from hemogenic endothelium in the floor of the dorsal aorta and subsequently migrate to secondary niches where they expand and differentiate into committed lineages. Glia maturation factor *γ* (*gmfg*) is a key regulator of actin dynamics that was shown to be highly expressed in hematopoietic tissue. Our goal is to investigate the role and mechanism of *gmfg* in embryonic HSPC development.

**Methods:**

In-depth bioinformatics analysis of our published RNA-seq data identified *gmfg* as a cogent candidate gene implicated in HSPC development. Loss and gain-of-function strategies were applied to study the biological function of *gmfg*. Whole-mount in situ hybridization, confocal microscopy, flow cytometry, and western blotting were used to evaluate changes in the number of various hematopoietic cells and expression levels of cell proliferation, cell apoptosis and hematopoietic-related markers. RNA-seq was performed to screen signaling pathways responsible for *gmfg* deficiency-induced defects in HSPC initiation. The effect of *gmfg* on YAP sublocalization was assessed in vitro by utilizing HUVEC cell line.

**Results:**

We took advantage of zebrafish embryos to illustrate that loss of *gmfg* impaired HSPC initiation and maintenance. In *gmfg*-deficient embryos, the number of hemogenic endothelium and HSPCs was significantly reduced, with the accompanying decreased number of erythrocytes, myelocytes and lymphocytes. We found that blood flow modulates *gmfg* expression and *gmfg* overexpression could partially rescue the reduction of HSPCs in the absence of blood flow. Assays in zebrafish and HUVEC showed that *gmfg* deficiency suppressed the activity of YAP, a well-established blood flow mediator, by preventing its shuttling from cytoplasm to nucleus. During HSPC initiation, loss of *gmfg* resulted in Notch inactivation and the induction of Notch intracellular domain could partially restore the HSPC loss in *gmfg*-deficient embryos.

**Conclusions:**

We conclude that *gmfg* mediates blood flow-induced HSPC maintenance via regulation of YAP, and contributes to HSPC initiation through the modulation of Notch signaling. Our findings reveal a brand-new aspect of *gmfg* function and highlight a novel mechanism for embryonic HSPC development.

**Supplementary Information:**

The online version contains supplementary material available at 10.1186/s13287-023-03328-1.

## Background

Hematopoietic stem and progenitor cells (HSPCs) give rise to all mature blood lineages and maintain self-renewal ability through the lifespan of an organism [1]. The production of bona fide HSPCs from pluripotent stem cells (PSCs) for therapeutic purposes has been a long-term pursuit in the field of hematopoiesis [2]. However, existing methods to derive or expand HSPCs in vitro remain inefficient, partially due to our incomplete knowledge on the molecular details underlying the development of naive embryonic hematopoietic stem cells (HSCs) [3]. Therefore, a full appreciation of the mechanisms regulating developing HSPCs would provide essential insights for the generation of HSPCs in vitro.

In vertebrate embryos, HSPCs specified from hemogenic endothelium (HE) via a stereotyped process termed endothelial-to-hematopoietic transition (EHT) [4]. With the aid of real-time imaging in zebrafish, scientists can clearly visualize the dynamic EHT process as flat-shaped HE bends, contracts in the direction of blood flow and dissociates from the aortic floor, until its eventual transformation into spherical HSPCs [5]. At the molecular level, the assembly of rings of actin and myosin proteins into anisotropic contractile circumferential actomyosin around stem cells was reported to promote this process [6, 7]. In recent decades, biomechanical signals relevant for the control of HSPC development, the best understood of which is blood flow, has emerged as one of the hot spots in the field of hematopoiesis [8]. Of interest, cytoskeletal architecture defects within EHT cells were observed followed by blood flow stagnation [6]. Recent studies in zebrafish have showed that Rho-GTPases, established molecular switches that control cytoskeleton dynamics [9], mediated blood flow-induced HSPC development via activation of Yes-activated protein (YAP)[10]. Moreover, defects in actin cytoskeleton remodeling also impaired lymphocyte development and activation[11]. Based on these evidence, actin cytoskeleton related elements and regulatory cascades were speculated to play an important role in hematopoiesis especially embryonic HSPC development.

Glia maturation factor *γ* (*gmfg*) is a 17-kDa protein that structurally belongs to the actin depolymerizing factor homology (ADF-H) family [12, 13]. All known ADF-H proteins are capable of binding to actin and/or actin-related proteins (ARPs) with a conserved role in cytoskeletal dynamics [14]. Two distinct *gmf* isoforms, *gmfb* and *gmfg*, which share 82% sequence conservation and significant similarity in their 3D structures, are expressed in vertebrates [13]. Unlike *gmfb* (brain-specific isoform), *gmfg* expression was not found in the brain, neuronal cells or glial cells, but was preferentially expressed in endothelial cells and inflammatory cells [15]. Functionally, *gmfg* has been established as a key regulator of cytoskeleton reorganization rather than glial maturation [15, 16]. Of interest, multiple studies have revealed that *gmfg* is closely associated with hematopoietic system. Accordingly, Shi et al. demonstrated that GMFG is a cytokine-responsive protein in erythropoietin (EPO)- and granulocyte-colony stimulating factor (G-CSF)-induced HSC differentiation in vitro. Global functional genomics analysis identified several high-score hematopoiesis-specific transcription factors binding sites in the *gmfg* promoter and putative molecular coevolution of *gmfg* with a rudimentary blood/immune system [17]. *gmfg* was thus presumed as a mediator of HSC pluripotency and lineage commitment, which however awaits to be proved by experiments. Regarding embryonic hematopoiesis, *gmfg* expression is abundant in the blood islands of the yolk sac and the DA [15]. In addition, genetic inhibition of *gmfg* attenuated the migration and/or chemotaxis of monocytes, neutrophils, T cells and B cells [18–22]. Another study reported that *gmfg* expression was significantly elevated in leukemia patients [23]. Nevertheless, whether and how *gmfg* regulates aspects of embryonic HSPCs have not been systematically explored. In this study, we uncovered an indispensable requirement for *gmfg* in the birth of embryonic HSPCs. Further data showed that *gmfg* is responsive to blood flow, which is essential for HSPC maintenance via regulation of Yap. Moreover, Notch was identified as a downstream effector of *gmfg* to control HSPC initiation, revealing a previously unappreciated role for this classical hematopoietic regulatory signaling during HSPC development.

## Methods

### Re-analysis of our published RNA-seq data

Package DESeq2 was used to calculate the upregulated differentially expressed genes (DEGs) in PSC-, fetal liver (FL)- and bone marrow (BM)-derived HSPCs as compared to undifferentiated PSCs. Foldchange > 2 and p.adjust < 0.05 were set as the cutoffs. Biological process enrichment analysis of the common upregulated DEGs was performed with the DAVID gene annotation tool (https://david.ncifcrf.gov/). The RNA-seq data are freely available online at GEO database (accession number: GSE175563).

### Zebrafish husbandry and strains

Zebrafish were purchased from the Chinese Zebrafish Resource Center in Wuhan, China, raised and maintained in fully automated zebrafish housing systems (Aquazone, Tzofit, Israel; temperature 28.5 °C, pH 7.0, conductivity 300 mS) under 14-h light/10-h dark cycles, in accordance with the relevant guidelines of the Laboratory Animal Center of Zhejiang University as described previously [24]. Adult zebrafish were fed with paramecium twice a day and larvae also were fed twice a day starting from 5 dpf. The embryos used in our study were all natural spawning of adult zebrafish at the age of 6–18 months, and raised in egg-water containing methylene blue (0.3 ppm) in a light-controlled incubator. For each set of experiments, the same clutches of embryos were randomly distributed for the control and experimental groups. An estimated 150 zebrafish adults were utilized in the study. Live embryos were anesthetized with 0.04‰ tricaine methanesulfonate (MS-222, the most widely used anaesthetic agent in aquatic species) for the subsequent experiments and were euthanized with 0.02% MS-222. All the procedures of the animal experiments were reviewed and approved by the Institutional Animal Care and Use Committee of the Laboratory Animal Center, Zhejiang University (Hang Zhou, China). Zebrafish strains used in our study have been listed in the Results and Figure legends section. Primers for genotyping of indicated strains are listed in Additional file 1: Table S1.

### Morpholino injections

Antisense morpholinos (MOs) used in our study were ordered from Gene Tools. Optimized working concentration of MOs was attained by diluting the MO powder in diethyl pyrocarbonate-treated water and phenol red solution, and 1 nl indicated MO was injected into 1 or 2 cell-stage embryos. The sequence of MOs used in this study are listed in Additional file 1: Table S2. Western blotting was performed to validate the specificity of *gmfg*-atgMO, *gmfg*-spMO and *gmfb*-MO (Additional file 1: Fig. S1a, b).

### mRNA synthesis and injections

For messenger RNA (mRNA) synthesis, total RNA was extracted from zebrafish wild type embryos using Trizol reagent (Invitrogen) and reverse transcribed into complementary DNA (Vazyme). Specific primers utilized to amplify the opening reading frame of *gmfg* are listed in Additional file 1: Table S3. The PCR product was cloned into the pCS2^+^ plasmid (Vazyme) and validated by bidirectional sequencing. The recombined pCS2^+^-*gmfg* plasmid was then linearized by NotI and purified (Takara Purification Kit). Capped full-length zebrafish *gmfg* mRNA was generated using SP6 mMESSAGE Kit (Life Technologies) according to the manufacturer’s instructions. Agar gel electrophoresis was performed to validate the *gmfg* mRNA band is correct in size (Additional file 1: Fig. S2a). 100 pg purified *gmfg* mRNA was injected into one-cell stage embryos alone or in combination with indicated MOs (*gmfg*-atgMO or tnnt2a-MO). Western blotting was carried out to validate the overexpression of *gmfg* (Additional file 1: Fig. S2b).

### Whole-mount RNA in situ hybridization

Whole-mount in situ hybridization (WISH) was performed as described [25]. Probes for *gmfg, runx1, cmyb, fli1a, efnb2a, dlc, mpx, l-plastin, rag1, foxn1* and *klf2a* transcripts were generated using a DIG RNA Labeling Kit (Roche Applied Science) from relevant linearized plasmids. Embryos were observed and photographed by a Leica M165C stereomicroscope equipped with a DFC295 color digital camera (Leica).

### Flow cytometry analysis and cell sorting

Cells derived from human embryonic stem cells (hESCs) at indicated time point of differentiation were harvested and suspended in phosphate-buffered saline (PBS) with 2% fetal bovine serum (FBS). Before antibody incubation, cells were blocked with an anti-CD16/32 antibody (eBioscience). Fluorescence-activated cell sorting (FACS) were based on the following markers: CD309-Percp/cy5.5 CD31-FITC CD34-APC, CD43-APC and CD45-PE (all kits Biolegend). DAPI was included to omit dead cells. Cells were stained in PBS/2% FBS for 30 min at room temperature and sorted out using BD AriaII (BD Biosciences, San Jose, CA). As for zebrafish, embryos were manually dechorionated or dechorionated with pronase, dissociated with liberase, and triturated using a P1000 pipette. The resulting single-cell suspension was filtered with a 40 μm cell strainer and resuspended in PBS. Flow cytometric acquisitions or FACS were performed on BD Fortessa and BD AriaII (BD Biosciences, San Jose, CA), respectively. Graphs were prepared in FlowJo.

### Quantitative reverse transcription polymerase chain reaction

Total RNA of FACS-sorted human cells was extracted using Trizol reagent (Invitrogen) and 1 μg RNA was reverse-transcribed into complementary DNA using HiScript II Q RT SuperMix for qPCR (+ gDNA wiper) according to the manufacturer’s instructions (Vazyme). As for zebrafish, FACS-sorted ECs or HE were collected and cDNA was directly generated using Single Cell Sequence Specific Amplification Kit (Vazyme) according to the manufacturer’s instructions, diluting three to five times as templates. Quantitative PCR (qPCR) was performed on a Bio-Rad CFX96 system using ChamQ Universal SYBR qPCR Master Mix (Vazyme) and samples were run in duplicate or triplicate with R3 biological replicate pools/condition using the primers listed in Additional file 1: Table S3. Relative expression was normalized to the human actin or zebrafish actb2 housekeeping gene.

### Western blot analysis

The trunk and tail (including AGM) of zebrafish embryos (50 or more) at a noted time point was dissected out after dechorionation, homogenized with deyolking buffer and washing buffer, and splitted in RIPA lysis buffer (Beyotime) containing protease inhibitor (Roche) for 2–4 h. Lysate was centrifuged and the resulting supernatant was added loading buffer as protein samples. After appropriate concentration of SDS-PAGE separation, proteins from zebrafish embryos or HUVEC were transferred to nitrocellulose membranes followed by blocking with 5% nonfat milk. After blocking, nitrocellulose membranes were incubated with indicated antibodies as listed in Additional file 1: Table S4. Membranes were washed in TBST and incubated with anti–mouse/rabbit secondary Ab (1:5000, Jackson ImmunoResearch Laboratories). After extensive washing, the target protein bands were detected by anti-ECL HRP. Signals from immunoreactive bands developed by using an ECL reagent and the intensity of individual bands in the immunoblots was visualized using the Image program (Clinx, Shanghai, China). The original, unprocessed scans of blots in the study were included in Additional file 2.

### Nuclear and cytoplasmic fractionation

Nuclear and cytoplasmic fractions were isolated using NE-PER Nuclear and Cytoplasmic Extraction Reagents (Thermo Scientific) following the manufacturer’s instructions. The levels of GAPDH and Lamin B1 were used as loading controls for the cytoplasmic and nuclear proteins, respectively.

### Confocal microscopy

Live *kdrl*:mCherry;*cmyb*:GFP double transgenic embryos, *fli1a*:eGFP*;tp1*:mCherry double transgenic embryos and *cd41*:eGFP transgenic embryos were anesthetized with 0.04‰ MS-222 and mounted carefully on dishes with 0.8% low-melting agarose. Confocal images of the Z sections of the DA or caudal hematopoietic tissue (CHT) region were captured by a FV1000 inverted laser scanning confocal microscope (Olympus). For red fluorescent proteins, the excitation wavelength was set at 561 nm, and the emitted light was collected between 575 and 620 nm; for green fluorescent proteins, the excitation wavelength was set at 488 nm, and the emitted light was collected between 490 and 590 nm; DAPI (for HUVEC cell line) was excited by 405 nm laser, and the emitted light was collected between 430 and 455 nm, all of the above using the PMT detector. Analysis of the images was carried out by Fluoview Ver.4.0b and Image J (https://imagej.nih.gov/ij/) software. The excitation laser power varied among distinct fluorescent proteins but remained constant throughout the experiments. The confocal image resolution is 485 × 485 pixels.

### Fluorescent visualization of HSPCs, neutrophils and erythroid cells

*cmyb*:GFP*, lcr*:*e*GFP*,* and *lyz:*dsRed transgenic embryos were used to visualize HSPCs, erythrocytes and neutrophils at indicated time points, respectively. They were anesthetized with 0.04‰ MS-222 and photographed by a Leica MZ16FA stereomicroscope. For red fluorescent proteins, the excitation wavelength was set at 561 nm. For green fluorescent proteins, the excitation wavelength was set at 488 nm. The fluorescent image resolution is 1608 × 1608 pixels.

### TUNEL assay and pH3 staining

TUNEL assay was carried out as described previously [24]. In brief, manually dechorionated *fli1a*:eGFP transgenic embryos were fixed in 4% PFA at 4 °C overnight, washed three times with PBST and dehydrated in 100% methanol at − 20 °C for more than 2 h. After gradual rehydration, embryos were washed with PBST three times followed by proteinase K and acetone treatment. After washing 3 times with PBST, the permeabilized embryos were incubated in a mixture containing labeling solution and enzyme solution at a ratio of 9:1 (In Situ Cell Death Detection Kit TMR Red, Roche) at 4 °C overnight. Finally, the embryos were washed three times with PBST and then were captured by confocal microscopy. pH3 staining was performed as follows: zebrafish embryos at noted time point were collected, fixed, dehydrated, rehydrated and permeabilized as TUNEL assay. After washing 3 times with PBST, embryos were blocked in the block buffer (Beyotime) for 1–2 h at room temperature and then stained with anti-PH3 (1:250, Abcam) and anti-GFP (1:1000, Life Technologies) at 4 °C overnight. Before imaging with the confocal microscopy, the embryos were washed 5 times with PBST and incubated with appropriate secondary antibodies [Alexa Fluor 488-conjugated anti-rabbit (1:1000, Life Technologies) and Alexa Fluor 647-conjugated anti-mouse (1:1000, Life Technologies)].

The embryos were mounted, imaged, and analyzed similarly to the confocal microscopy protocol outlined above. The image resolution is 586 × 586 pixels.

### Chemical treatment

Zebrafish embryos were exposed to chemicals at indicated concentration, dimethyl sulfoxide (DMSO) carrier content was 0.1%. For evaluation of HSPC development, exposure ranged from 12 hpf until fixation and subjected to WISH for *runx1*/*cmyb*. Siblings treated with 0.1% DMSO in Hotter buffer were taken as controls. Detailed information of chemicals utilized in this work is compiled and listed in Additional file 1: Table S5.

### Heat-shock treatment

For induction of *hsp70l*:Gal4-driven NICD overexpression, embryos were placed in E3 medium and transferred to a 37 °C cell incubator for 30 min at noted stages.

### RNA sequencing

Total RNA was extracted from the dissected trunk and tail (including AGM) of 26 hpf *gmfg* morphants and their control siblings using TRIzol Reagent (Invitrogen, Carlsbad, CA, USA) according to the manufacturer’s instructions, and was further qualified and quantified using a Nano Drop and Agilent 2100 Bioanalyzer (Thermo Fisher Scientific, Waltham, MA, USA). Afterwards, oligo (dT)-attached magnetic beads were employed to purify mRNA. Purified mRNA was fragmented into small pieces, followed by a sequential first & second-strand cDNA synthesis. Subsequently, a-Tailing Mix and RNA Index Adapters were added by incubating to end repair. PCR was performed to amplify the cDNA fragments acquired from previous step and products were purified by Ampure XP Beads. Then, the double-stranded PCR products were verified, heated denatured and circularized to gain the final library in single strand circle DNA (ssCir DNA) form. DNA nanoballs (DNBs) comprising more than 300 copies of one molecule were obtained by amplifying the final library with phi29 and then were loaded into the patterned nanoarray. Single end 50 bases reads were produced on BGIseq500 platform (BGI-Shenzhen, China).

The sequencing data was processed and filtered with SOAPnuke (v1.5.2). Clean reads were acquired and mapped to the reference genome (GRCz11) using HISAT2 (v2.0.4). Bowtie2 (v2.2.5) was employed to align the clean reads to the reference coding gene set. To explore the mechanisms underlying the change of phenotype, gene set enrichment analyses (GSEA) was performed using the software gsea-3.0 downloaded from Broad Institute. The whole transcriptome of all samples was used for GSEA, and |NES (normalized enrichment score)|> 1 and nominal *p*-value < 0.05 were considered statistically significant.

### Statistical analysis

Statistical analysis was performed using the GraphPad software (Prism 8). Normal distribution of the data was tested by using the D’Agostino-Pearson test. If the data showed normal distribution, a two sample t test was performed to compare two independent groups (Welch’s t test). For comparison of multiple groups showing normal distribution, a one-way ANOVA was used with multiple comparison correction using Dunnett’s T3 multiple comparison test. Sample sizes were indicated in the figures or figure legends. In all statistical figures, solid red bars denote the mean, and error bars represent the SD/SEM. If not stated otherwise, data is presented as mean ± SD/SEM value. *P < 0.05 was considered statistically significant, **p < 0.01, ***p < 0.001, ****p < 0.0001; n.s., not significant.

## Results

### *gmfg* is a potential regulator of HSPC development

**Fig. 1:**
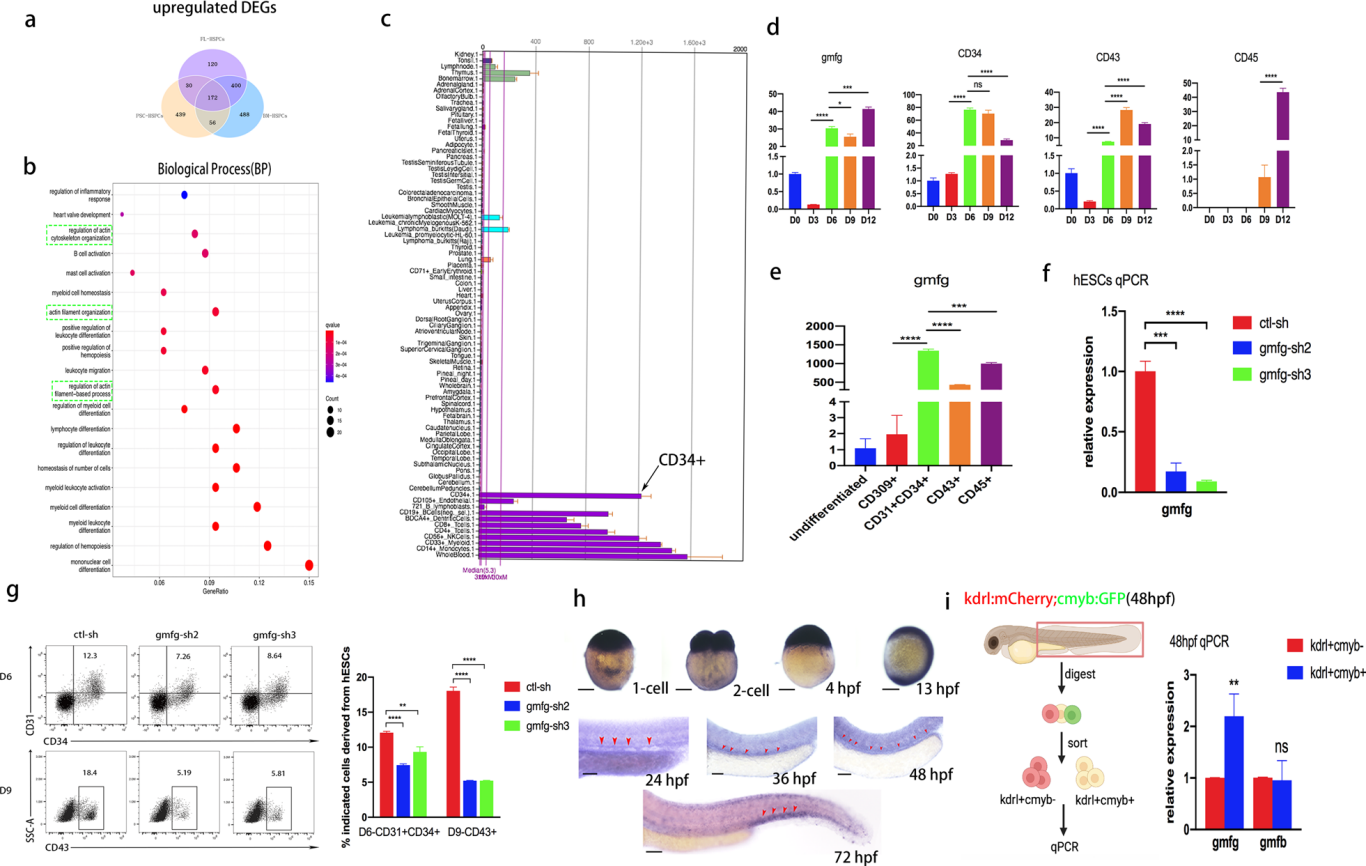
*gmfg* is a potential regulator of HSPC development. **a** Venn diagram showing the upregulated DEGs in the PSC-, FL- and BM-derived HSPCs as compared to undifferentiated PSCs (172 common upregulated DEGs). **b** Top 20 statistically enriched pathways of the common upregulated DEGs in the PSC-, FL- and BM-derived HSPCs. **c** *gmfg* expression in human tissues. The abscissa represents different human tissues, the ordinate represents gene expression level, and the histogram represents the expression level of *gmfg* in specific human tissue. Black arrow denotes *gmfg* expression in CD34 + cells. **d** Dynamic analysis of *gmfg*, CD34, CD43 and CD45 expression with real-time PCR during hESC hematopoietic differentiation. Relative expression is normalized by undifferentiated hESCs (except for CD45 by differentiated hESCs at D9). Data are shown as mean ± SD (*n* = 3). ns, not significant; *p < 0.05, ***p < 0.001, ****p < 0.0001. **e** Quantitative RT-PCR analysis of *gmfg* expression in undifferentiated hESCs, mesoderm (CD309 +), HEPs (CD31 + CD34 +), and HPCs (CD43 + and CD45 +) derived from hESCs. Relative expression was normalized by undifferentiated hESCs. Data are shown as mean ± SD (*n* = 3). ***p < 0.001, ****p < 0.0001. **f** Quantitative RT-PCR analysis of *gmfg* expression in hESCs treated with ctl-sh, *gmfg*-sh2, and *gmfg*-sh3. Data are shown as mean ± SD (*n* = 3). ***p < 0.001, ****p < 0.0001. **g** Flow cytometry analysis of CD31 + CD34 + HEPs and CD43 + HPCs generated from ctl-sh, *gmfg*-sh2, and *gmfg*-sh3 hESCs at day6 and day9 of differentiation, respectively. Data are shown as mean ± SD (*n* = 3). **p < 0.01, ****p < 0.0001. **h** Expression pattern of *gmfg* during zebrafish embryogenesis: The stage examined by whole-mount in situ hybridization (WISH) is shown in each panel: 1‐cell stage, 2‐cell stage, 4 h postfertilization (hpf), 13 hpf (Scale bars: 200 μm), 24 hpf, 36 hpf, 48 hpf and 72 hpf (Scale bars: 100 μm). **i** (left) Graphic representation of the sorting strategy of *kdrl* + *cmyb*- ECs and *kdrl* + *cmyb* + HE from *kdrl*:mCherry;*cmyb*:GFP transgenic zebrafish by FACS at 48 hpf. (right) Quantitative RT-PCR analysis of *gmfg* and *gmfb* expression in FACS-sorted *kdrl* + *cmyb*- ECs and *kdrl* + *cmyb* + HE. Bars represent mean ± SD (*n* = 3). ns, not significant; **p < 0.01

Endothelial protein C receptor (EPCR), also known as CD201, has been identified as a novel HSC marker in mouse embryos and human adults [26–29]. Very recently we established a 3D induction system cocultured with stromal cells, capable of yielding a higher percentage of CD201 + HSC-like cells (Lin-Sca-1 + c-kit + CD201 + , LSKCD201 +) with robust hematopoietic reconstitution potential from mouse PSCs [30], which we defined as PSC-derived HSPCs hereafter. By taking advantage of this model, we previously performed RNA sequencing on mouse undifferentiated PSCs, PSC-derived HSPCs, FL-derived HSPCs (FL-LSKCD201 +), and BM-derived HSPCs (BM-LSKCD201 +) [30]. To dig into the biological generalities and screen potential regulators implicated in HSPC development, we re-analyzed the RNA-seq data and supposed that genes upregulated in HSPCs must play a critical role. According to a rigorous cut-off criterion (Foldchange > 2 and p.adjust < 0.05), 697, 722 and 1116 upregulated DEGs in PSC-, FL- and BM-derived HSPCs as compared to undifferentiated PSCs were identified, respectively. Common upregulated DEGs were defined as genes that were significantly upregulated in all the three populations and thereby 172 genes were discovered (Fig. 1a). Further application of biological process enrichment analysis revealed that these common upregulated DEGs were strongly linked to actin cytoskeleton organization, with 20 related genes involved (Fig. 1b). Afterwards, by searching the public database of BIOGPS (http://biogps.org/#goto=welcome), we found that *gmfg* was most prominently expressed in the human blood system, and was much more highly expressed in CD34 + HSCs than in their mature counterparts (NK cells, B cells and T cells) (Fig. 1c), indicating a more important role of *gmfg* in HSCs than in the precursor/mature stages. To confirm the data from RNA-Seq and public databases and determine the exact time window when *gmfg* began to work during hematopoietic development, we performed real-time PCR analysis as hESCs progressed through hematopoietic differentiation. Results showed that *gmfg* expression increased sharply at day 6 (D6) of differentiation, and maintained high-level expression as maturation towards hematopoiesis. The expression of CD34, CD43 and CD45, three well-known HSPC markers representing distinct phases of differentiation, was detected to validate that the genetic program for hematopoiesis in our system is activated at D6 (Fig. 1d). Furthermore, stage-specific cell populations at various time points were purified to document the cell-type specificity of *gmfg.* Consistently, *gmfg* exhibited a greater than 600-fold upregulation in D6-CD31 + CD34 + hemogenic endothelium progenitors (HEPs) as compared to D3-CD309 + mesoderm cells, and then it was slightly downregulated in D9-CD43 + and D12-CD45 + hematopoietic progenitor cells (HPCs) (Fig. 1e). Therefore, the stage of HEP specification from mesoderm cells was proposed as the appropriate time window that *gmfg* began to play a role. For illustration of the idea, we evaluated the impact of *gmfg* deletion on the generation of HEPs and HPCs from hESCs by utilizing a short hairpin RNA oligonucleotides (shRNA)-mediated *gmfg* knockdown approach. The sequence of control and *gmfg* shRNAs was listed in Additional file 1: Table S6. *gmfg*-sh2 and *gmfg*-sh3 were chosen as the main tools for the next experiments since the mRNA level of *gmfg* was remarkably decreased in hESCs treated with these two shRNAs (Fig. 1f). As expected, flow cytometry analysis showed that *gmfg* deletion significantly reduced the fraction of D6-CD31 + CD34 + HEPs and subsequent D9-CD43 + HPCs derived from hESCs (Fig. 1g), confirming the impairment of hESC hematopoietic differentiation.

Since hematopoietic differentiation of PSCs in vitro well recapitulates embryonic hematopoiesis in vivo [31], above evidence prompted us to further probe the role of *gmfg* in embryonic hematopoiesis. We therefore analyzed the dynamic expression of *gmfg *in vivo by WISH during zebrafish embryogenesis. Though ubiquitously expressed from one-cell stage to 13 h postfertilization (13 hpf), *gmfg* mRNA exhibited a marked enrichment in the AGM from 24 to 48 hpf, and then it was found in the CHT at 72 hpf (Fig. 1h). This specific spatio-temporal expression pattern was reminiscent of the developmental trajectory of embryonic HSPCs. Consistently, *kdrl* + *cmyb-* endothelial cells (ECs) and *kdrl* + *cmyb* + HE were sorted from 48 hpf *kdrl*:mCherry;*cmyb*:GFP embryos and qPCR was performed for *gmfg* and *gmfb*, another *gmf* isoform. Compared to ECs, *gmfg* expression was much higher in the developing HE, whereas *gmfb* expression was comparable in both groups of cells (Fig. 1i). Altogether, these data strongly suggested that *gmfg* is a potential regulator of HSPC development.

### *gmfg*, instead of *gmfb*, is required for HSPC development

To investigate whether *gmfg* has a function in HSPC development, loss-of-function experiments were performed by utilizing two different types of antisense morpholino oligonucleotides (MOs) against *gmfg*, namely, translation blocking MO (*gmfg*-atgMO) and splice modifying MO (*gmfg*-spMO). The efficiency of these two MOs was validated by western blot analysis, in which both MOs could significantly reduce the protein level of *gmfg* at 36 hpf and 4 dpf, respectively (Additional file 1: Fig. S1a), and *gmfg*-atgMO was chosen as the main tool for subsequent experiments. In particular, of the reference genes described in various publications, β-actin and GAPDH were the most widely used ones. However, previous studies have reported that GAPDH expression varies during zebrafish embryonic development and can't be detected at early stages (no detectable until the Prim 5 stage), rendering it unsuitable as an internal control for zebrafish developmental time course studies [32]. In our study, Actin (β-actin) and GAPDH were simultaneously subjected to western blot analysis and results showed that no significant changes in the protein level of Actin compared with Gapdh was observed between control and *gmfg* morphants at 48 hpf (Additional file 1: Fig. S3), demonstrating that *gmfg* deficiency did not grossly affect the expression stability of Actin. Therefore, though *gmfg* is a regulator in actin dynamics, β-actin is still considered to be superior to GAPDH for normalization in this study.

**Fig. 2:**
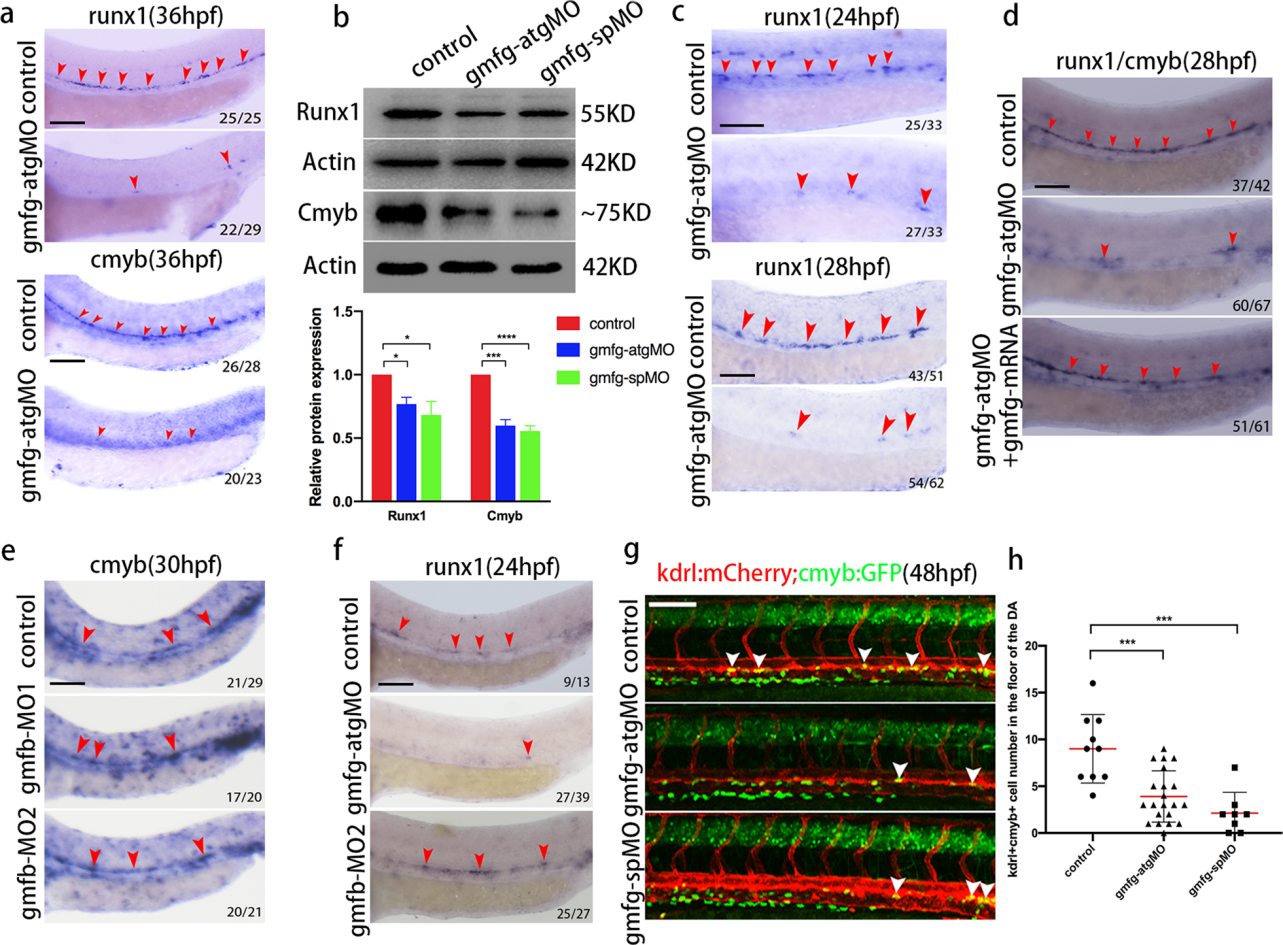
Loss of *gmfg*, but not *gmfb*, induces HSPC defects. **a** WISH results of *runx1* and *cmyb* in the AGM in control and *gmfg* morphants at 36 hpf. The red arrowheads indicate the expression of *runx1* and *cmyb*. **b** Western blotting showing the protein level of Runx1 and Cmyb in control and *gmfg* morphants at 36 hpf. Representative blot is shown in the figure (Full-length blots are presented in Additional file 2: Fig. S1 and S2). Data represent mean ± SEM intensity of indicated blots (*n* ≥ 3). *p < 0.05, ***p < 0.001, ****p < 0.0001. **c** WISH analysis showing the expression of *runx1* (red arrowheads) in the DA in control and *gmfg* morphants at 24 and 28 hpf. **d** WISH analysis showing *runx1/cmyb* expression (red arrowheads) in the DA in control, *gmfg*-atgMO, and coinjection of *gmfg*-atgMO and *gmfg* mRNA embryos at 28 hpf. **e** WISH analysis showing the expression of *cmyb* (red arrowheads) in the DA in embryos injected with control MO and *gmfb*-MO at two different doses (*gmfb*-MO1: 9.6 ng and *gmfb*-MO2: 12.8 ng per embryo) at 30 hpf. **f** Representative images showing *runx1* expression (red arrowheads) in the DA in control, *gmfg*-atgMO and *gmfb*-MO2 (12.8 ng per embryo) at 24 hpf. **g** Maximum projections of 48 hpf *kdrl*:mCherry; *cmyb*:GFP double-transgenic embryos injected with control MO, *gmfg*-atgMO, and *gmfg*-spMO. Arrowheads denote *kdrl* + *cmyb* + HE along the DA. All views: anterior to left. **h** Enumeration of *kdrl* + *cmyb* + HE shown in (g). Bars represent mean ± SD of control MO (*n* = 10), *gmfg*-atgMO (*n* = 21), and *gmfg*-spMO (*n* = 8). ***p < 0.001. Numbers at the lower right corner of the picture represent embryos with displayed phenotype/whole embryos. All scale bars, 100 µm

In zebrafish embryos, HSPCs can be visualized along the axial vessels by the expression of *runx1* and *cmyb*, two conserved HSPC markers. *runx1* + and *cmyb* + HSPCs in the DA were dramatically decreased or even absent in the embryos injected with *gmfg*-atgMO at 36 hpf (Fig. 2a). This result was supported by western blot analysis of the protein levels of Runx1 and Cmyb, which were also significantly reduced in embryos injected with *gmfg*-atgMO, and *gmfg*-spMO phenocopied the *gmfg*-atgMO results (Fig. 2b). These reductions could be due to a defect in the earliest steps of HSPC initiation, therefore, we analyzed the nascent HSPC marker *runx1* expression at earlier time points. A significant reduction in the number of *runx1* + HSPCs was observed in *gmfg*-deficient embryos in or near the floor of DA at 24 and 28 hpf (Fig. 2c), indicating that *gmfg* plays an important role in HSPC initiation. To determine whether the HSPC loss observed in *gmfg* morphants resulted from growth retardation, we carried out time course analysis of *cmyb* expression at later phases. WISH and fluorescence imaging results showed that *cmyb* expression was always decreased in the DA and CHT in *gmfg* morphants at 48, 52 and 75–77 hpf, respectively (Additional file 1: Fig. S4a, b). To further confirm that HSPC loss was indeed specific to *gmfg*, we overexpressed *gmfg* by injecting the full-length zebrafish *gmfg* mRNA (escaping from *gmfg*-atgMO targeting) into embryos at one-cell stage to perform rescue experiments. The specificity of *gmfg* mRNA was validated by gel electrophoresis and western blot analysis (Additional file 1: Fig. S2a, b). WISH results showed that coinjection of *gmfg* mRNA with *gmfg*-atgMO was sufficient to normalize the decreased *runx1*/*cmyb* transcript levels in embryos injected with *gmfg*-atgMO alone at 28 hpf (Fig. 2d).

To determine whether *gmfb* also plays a role in HSPC development, we injected *gmfb*-MO into embryos at different doses and performed WISH analysis of *runx1* and *cmyb*. *gmfb*-MO at either dose, including a moderate amount of 9.6 ng (*gmfb*-MO1) and a maximum amount of 12.8 ng (*gmfb*-MO2) which caused slight malformation of embryos, showed no effect on the expression of *cmyb* at 30 hpf (Fig. 2e). Moreover, we injected *gmfb*-MO2 into zebrafish embryos again, and 1 ng *gmfg*-atgMO was used as a positive control, to detect *runx1* expression in the DA at 24 hpf. *runx1* expression remained unchanged in embryos injected with *gmfb*-MO2 but significantly decreased in embryos injected with 1 ng *gmfg*-atgMO (Fig. 2f). These results indicated that *gmfg*, but not *gmfb*, is required for HSPC development. We therefore focused on *gmfg* and *gmfb* was not pursued further in this study. In addition, we directly visualized the emerging HE undergoing EHT in the aortic floor using living *kdrl*:mCherry;*cmyb*:GFP double transgenic embryos by confocal microscopy. The number of *kdrl* + *cmyb* + HE in the floor of the DA was reduced by approximately 50%-70% in *gmfg* morphants in comparison to their control siblings (Fig. 2g, h), suggesting that the EHT process was severely impeded.

**Fig. 3:**
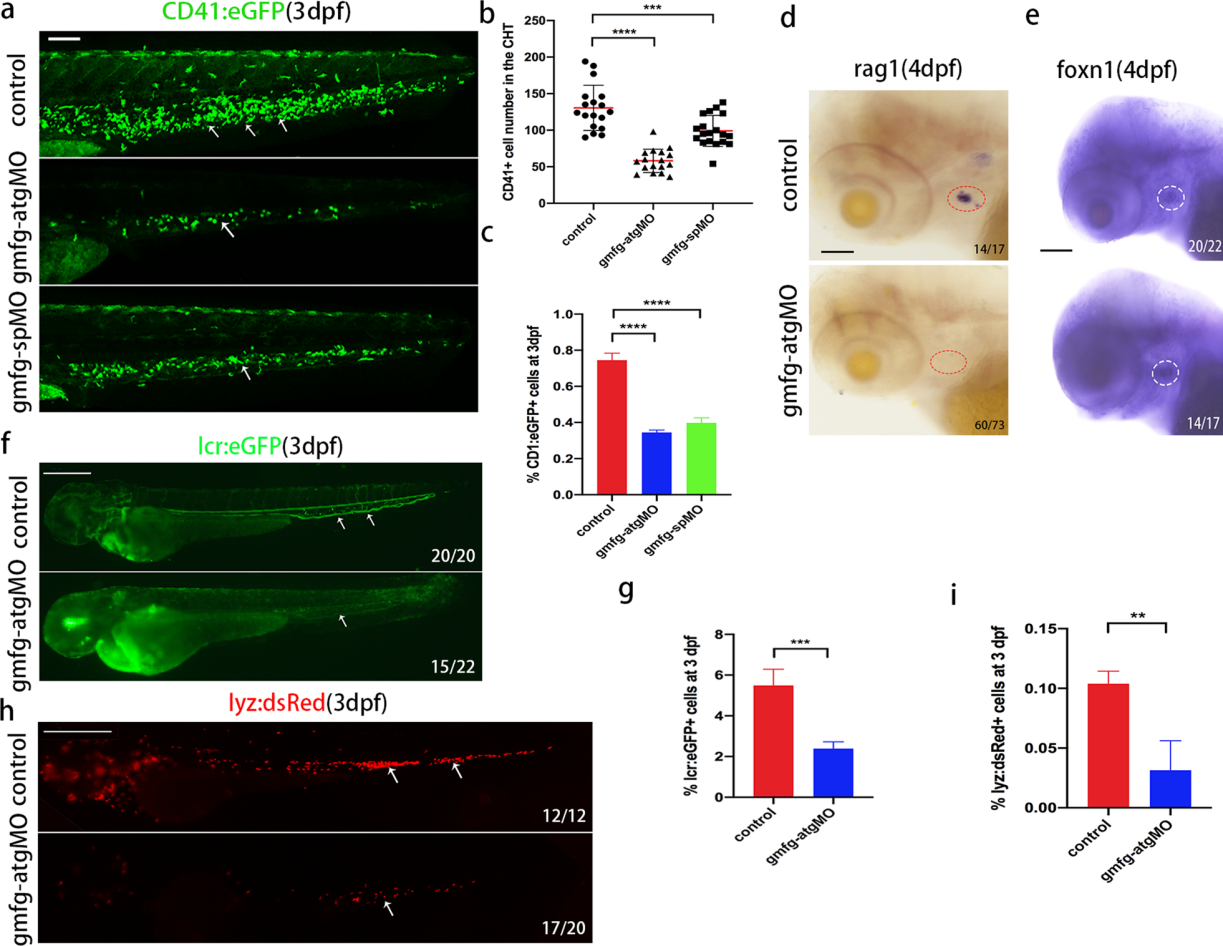
Loss of *gmfg* impairs the differentiation potential of HSPCs. **a** Maximum projections of 3 dpf *cd41*:eGFP transgenic embryos injected with control MO, *gmfg*-atgMO, and *gmfg*-spMO. White arrowheads denote *cd41* + cells in the CHT. All views: anterior to left. Scale bar, 100 μm. **b** Enumeration of *cd41* + cells shown in (a). Bars represent mean ± SD of control (*n* = 18), *gmfg*-atgMO (*n* = 17), and *gmfg*-spMO (*n* = 18). ***p < 0.001, ****p < 0.0001. **c** FACS analysis showing the percentage of *cd41* + cells in the dissected trunk and tail of control, *gmfg*-atgMO, and *gmfg*-spMO at 3 dpf (n = 3). ****p < 0.0001. **d** and **e** WISH for the T lymphocyte marker *rag1* (left, red dotted line circle) and thymic epithelial marker *foxn1* (right, white dotted line circle), respectively, in embryos injected with control MO and *gmfg*-atgMO at 4 dpf. All views are lateral, with anteriors to left. Scale bars, 50 µm. **f** and **h** *lcr*:eGFP and *lyz*:dsRed transgenic embryos injected with control MO and *gmfg*-atgMO were visualized at 3 dpf. Scale bars, 500 μm. **g** and **i** FACS analysis showing the percentage of *lcr* + erythrocytes and *lyz* + neutrophils in whole embryos of control and *gmfg*-atgMO groups at 3 dpf (*n* = 3). **p < 0.01, ***p < 0.001. Numbers at the lower right corner of the picture represent embryos with displayed phenotype/whole embryos

HSPCs produced by EHT in zebrafish subsequently migrate to and expand in the CHT which mirrors the functions of fetal liver in mammals [33]. We then utilized *cd41*:eGFP transgenic animals to track the development of HSPCs in the CHT. Confocal microscopy results revealed that the number of *cd41* + cells (*cd41*^low^ HSPCs and *cd41*^high^ thrombocytes) was markedly reduced in *gmfg*-deficient larvae at 3 dpf (Fig. 3a, b), which was further verified by flow cytometry quantitation of *cd41* + cells from the dissected trunk and tail (Fig. 3c). Furthermore, since T cells exclusively originate from definitive HSPCs, we examined later larval stages by detecting the expression of *rag1*, a T lymphocyte-specific marker. The transcript level of *rag1* was nearly or even completely absent in the *gmfg*-deficient larvae at 4 dpf (Fig. 3d), whereas the thymic anlage developed normally as assessed by the expression of a thymic epithelial cell marker *foxn1* (Fig. 3e). Moreover, *lcr*:eGFP and *lyz*:dsRed transgenic animals were utilized to track the developing erythrocytes and neutrophils. Fluorescence imaging and flow cytometry results showed that *gmfg* deficiency led to a significantly decrease in the number and percentage of erythrocytes/*lcr*:eGFP cells (Fig. 3f, g) and neutrophils/*lyz*:dsRed cells (Fig. 3h, i) at 3dpf, respectively, suggesting that to a certain extent, the differentiation potential of HSPCs into erythroid and myeloid lineages was compromised in *gmfg* morphants, although primitive hematopoiesis- and erythromyeloid progenitor (EMPs)-derived erythrocytes and neutrophils cannot be entirely excluded.

A widely accepted concept in embryonic hematopoiesis is that at least two waves of hematopoiesis occurred, namely primitive and definitive wave, which take place in anatomically distinct locations at different developmental time and can be further distinguished on the basis of cell types produced [34]. We next investigated whether *gmfg* is required for the initial wave of hematopoiesis commonly termed ‘primitive hematopoiesis’ due to the absence of upstream multipotent progenitors. In zebrafish, primitive hematopoiesis produces myeloid cells and erythrocytes that maintain early immunity and oxygenation [35]. The number of primitive erythrocytes in the intermediate cell mass (ICM) at 24 hpf was significantly decreased in the *gmfg*-deficient animals, as assayed by utilizing transgenic *lcr*:eGFP embryos (Additional file 1: Fig. S5a), and the expression of panleukocyte marker *l-plastin* and neutrophil marker *mpx* in the anterior lateral mesoderm (ALM) and the posterior lateral mesoderm (PLM) at 28 hpf was also markedly reduced (Additional file 1: Fig. S5b, c), suggesting that *gmfg* also is indispensable for primitive hematopoiesis. However, as primitive hematopoiesis is not the focus of our study, a more detailed role and mechanism of *gmfg* in primitive hematopoiesis is not explored further.

### *gmfg* does not appear to modulate PLM formation, DA specification, and EC proliferation or apoptosis

**Fig. 4:**
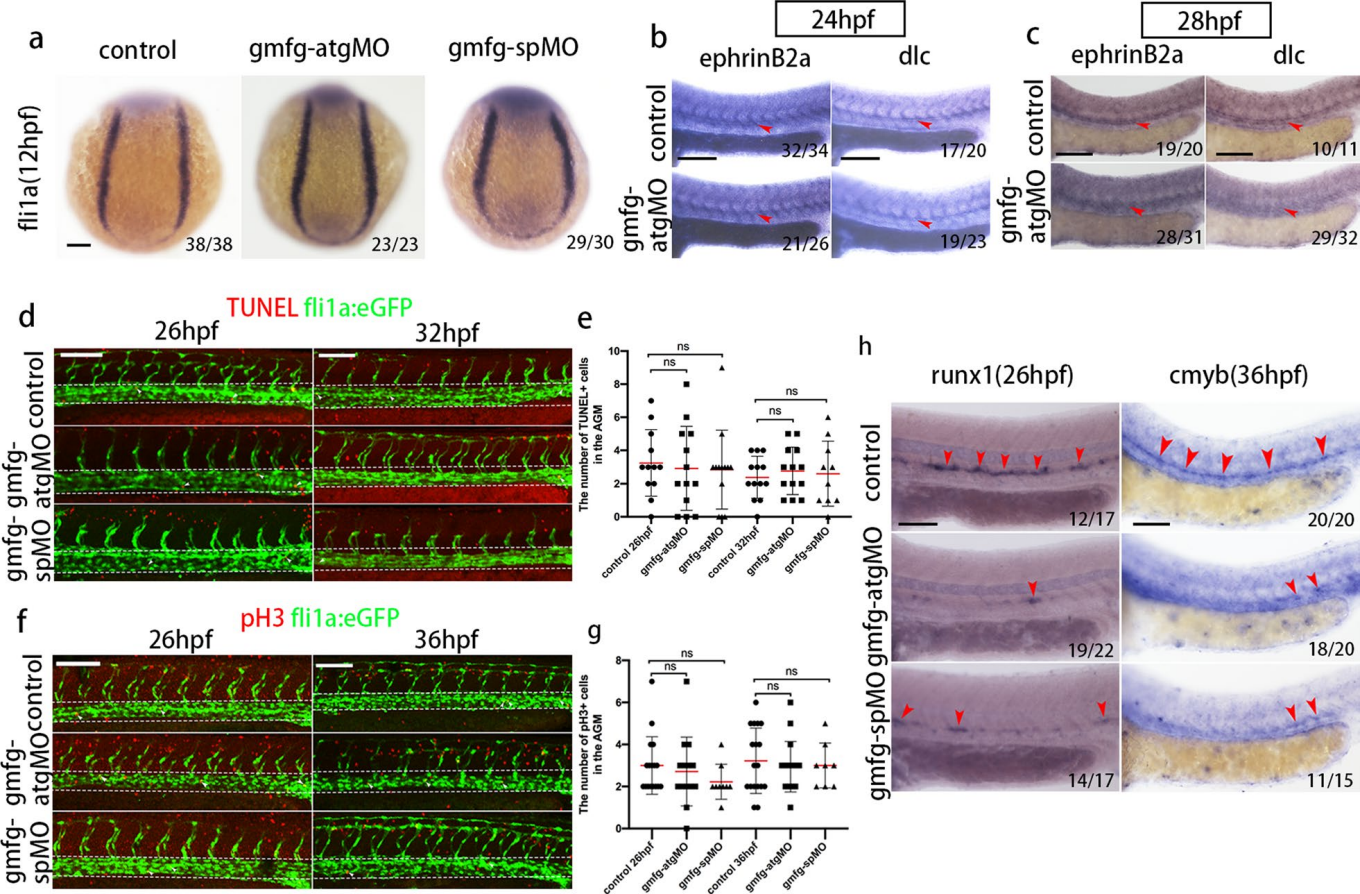
The effect of *gmfg* deficiency on PLM formation, DA specification, and EC proliferation or apoptosis. **a** Expression of the PLM marker *fli1a* in embryos injected with control MO, *gmfg*-atgMO, and *gmfg*-spMO analyzed by WISH at 12 hpf. **b** and **c** Expression of the DA-specific markers *ephrinB2a* and *dlc* in control and *gmfg* morphants analyzed by WISH at 24 (b) and 28 hpf (c). **d** TUNEL staining in *fli1a*:eGFP transgenic embryos injected with control MO, *gmfg*-atgMO, and *gmfg*-spMO at 26 and 32 hpf. White dashed lines indicate blood vessels and white arrowheads indicate the apoptosis of *fli1a* + cells. **e** Quantification of apoptotic *fli1a* + TUNEL + cells in (d). Bars represent mean ± SD of control (*n* = 18), *gmfg*-atgMO (*n* = 17), and *gmfg*-spMO (*n* = 18). ns, not significant. **f** PH3 staining in *fli1a*:eGFP transgenic embryos injected with control MO, *gmfg*-atgMO, and *gmfg*-spMO at 26 and 36 hpf. White dashed lines indicate blood vessels and white arrowheads indicate proliferative *fli1a* + cells. **g** Quantification of proliferative *fli1a* + pH3 + cells in (f), Bars represent mean ± SD of control (*n* = 18), *gmfg*-atgMO (*n* = 17), and *gmfg*-spMO (*n* = 18). ns, not significant. **h** Expression of *runx1* and *cmyb* in the AGM in p53 mutant injected with control MO, *gmfg*-atgMO, and *gmfg*-spMO at 26 (left) and 36 hpf (right). The red arrowheads mark HSPCs in the AGM. Numbers at the lower right corner of the picture represent embryos with displayed phenotype/whole embryos. All scale bars, 100 µm

Posterior lateral mesoderm (PLM) produces both endothelial and hematopoietic lineages [36]. To rule out the possibility that the observed HSPC defects upon *gmfg* deficiency were a consequence of impaired earlier PLM formation, we analyzed the expression of PLM marker *fli1a* at 12 hpf. Results showed that *fli1a* expression in *gmfg* morphants remained normal when compared to that of control siblings (Fig. 4a), demonstrating that PLM formation was unaffected. Because HSPCs derive from DA and many mutants with impaired arterial specification also display defective hematopoiesis [37], we assessed whether *gmfg* was required for DA specification. Knockdown of *gmfg* did not yield any obvious arterial abnormalities at 24 hpf, as evident from the unchanged expression of two DA-specific markers *ephrinB2a* and *dlc *[38] in embryos injected with *gmfg*-atgMO at the doses used in this study (Fig. 4b). However, as embryos developed at the late stage of 28 hpf, the expression of *ephrinB2a* and *dlc* within the DA was significantly reduced (Fig. 4c), indicating *gmfg* is dispensable for DA specification but is required for later DA development.

To probe whether HSPC loss observed in *gmfg* morphants could be attributed to abnormal apoptosis or proliferation of ECs, we performed TUNEL assay and immunostaining for proliferative marker PH3 using *fli1a*:eGFP transgenic animals[39]. Analysis of ECs by confocal microscopy showed that no significant difference in cell proliferation or apoptosis was present in the AGM between *gmfg* morphants and their control counterparts (Fig. 4d–g). Injection of antisense MOs often induces a nonspecific p53-dependent apoptosis [40], to address this issue, *gmfg*-atgMO and *gmfg*-spMO were injected into the p53 mutant at one-cell stage. The expression of *runx1* and *cmyb* was still reduced in *gmfg* morphants (Fig. 4h), suggesting that the HSPC loss was not caused by p53 activation. Altogether, these observations support the notion that *gmfg* regulates HSPC development independently of its role in PLM formation, DA specification, and EC proliferation or apoptosis.

### Blood flow-regulation of HSPC development is mediated, in part, by *gmfg*

**Fig. 5:**
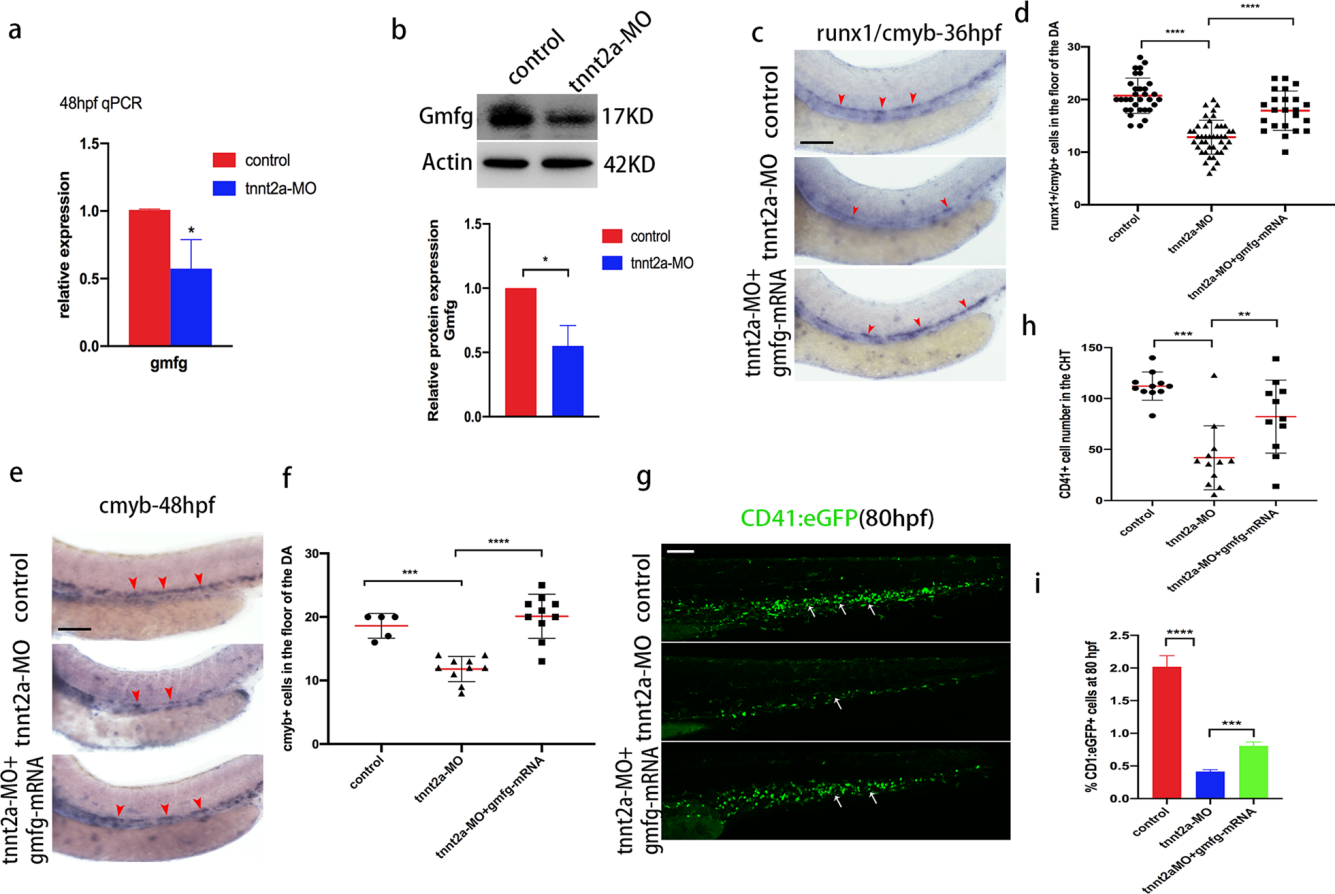
*gmfg* mediates blood flow-dependent HSPC development. **a** Quantitative RT-PCR analysis of *gmfg* expression in FACS-sorted *kdrl* + *cmyb* + HE of control and *tnnt2a*-MO embryos at 48 hpf. Bars represent mean ± SD (*n* = 3). *p < 0.05. **b** Western blotting showing the protein level of Gmfg in the dissected trunk and tail of control and *tnnt2a*-MO embryos at 48 hpf. Representative blot is shown in the figure (Full-length blots are presented in Additional file 2: Fig. S3). Data represent mean ± SEM intensity of indicated blots (*n* = 3). *p < 0.05. **c** Representative images showing *runx1*/*cmyb* expression (red arrowheads) in the DA in control, *tnnt2a*-MO, and coinjection of *tnnt2a*-MO and *gmfg* mRNA embryos at 36 hpf. **d** Enumeration of *runx1* + /*cmyb* + HSPCs shown in (c). Bars represent mean ± SD of control (*n* = 32), *tnnt2a*-MO (*n* = 43), and *tnnt2a*-MO + *gmfg*-mRNA (*n* = 23) embryos. ****p < 0.0001. **e** Representative images showing *cmyb* expression (red arrowheads) in the DA of control, *tnnt2a*-MO, and coinjection of *tnnt2a*-MO and *gmfg* mRNA embryos at 48 hpf. **f** Enumeration of *cmyb* + HSPCs shown in (e). Bars represent mean ± SD of control (*n* = 5), *tnnt2a*-MO (*n* = 10), and *tnnt2a*-MO + *gmfg*-mRNA (*n* = 10) embryos. ***p < 0.001, ****p < 0.0001. **g** Maximum projections of 80 hpf *cd41*:eGFP transgenic embryos injected with control MO, *tnnt2a*-MO, and *tnnt2a*-MO + *gmfg*-mRNA. Arrowheads denote *cd41* + cells in the CHT. All views: anterior to left. **h** Enumeration of *cd41* + cells shown in (g). Bars represent mean ± SD of control (*n* = 11), *tnnt2a*-MO (*n* = 12), and *tnnt2a*-MO + *gmfg*-mRNA (*n* = 11) embryos. **p < 0.01, ***p < 0.001. **i** FACS analysis showing the percentage of *cd41* + cells in the dissected trunk and tail of control, *tnnt2a*-MO, and *tnnt2a*-MO + *gmfg*-mRNA embryos at 80 hpf (*n* = 3). ***p < 0.001, ****p < 0.0001. All scale bars, 100 µm

Since we did not find evidence for the involvement of *gmfg* in the proliferation or survival of HSPCs in the AGM, we then turned our attention to the cellular mechanisms underlying HSPC reduction upon loss of *gmfg*. Considering the main function of GMFG in actin dynamics through its actin-severing/depolymerizing activities [13], we determined to examine the subcellular localization of GMFG and its colocalization with F-actin in HUVEC. Results showed that GMFG localized preferentially in the F-actin-rich structures at the forward periphery of cells as well as in cytoplasm and the colocalization of GMFG with F-actin was observed in these two regions (Additional file 1: Fig. S6), implying its putative role in actin cytoskeleton turnover by severing/depolymerizing filaments. On the other hand, elegant work from Lancino et al. demonstrated that blood flow regulated the morphodynamics of EHT-undergoing cells during HSC emergence [6], we therefore hypothesized that *gmfg* might mediate blood flow-dependent HSPC development through regulation of actin cytoskeleton reorganization. To validate this notion, we monitored *gmfg* expression variations in silent heart (*sih*/*tnnt2a*) embryos lacking a heartbeat and blood circulation [41]. Firstly, we constructed a silent heart model by using a previously verified *tnnt2a*-MO [42]. In comparison with their control siblings (Additional file 3), heart beating and blood flow were arrested and even invisible in *tnnt2a* morphants at 48 hpf as assessed by utilizing a *lcr*:eGFP transgenic line that can track the erythrocytes circulating in blood vessels (Additional file 4). HE was then isolated from control and *tnnt2a* morphants to perform qPCR for *gmfg*. As shown in Fig. 5a, the mRNA level of *gmfg* was significantly reduced in *tnnt2a* morphants and that this reduction was further confirmed by western blot analysis of Gmfg from dissected trunk and tail (Fig. 5b). Both results suggested that *gmfg* could well respond to hemodynamic alterations and justified *gmfg* as a downstream target of blood flow.

Next we sought to determine whether *gmfg* induction could restore HSPC loss in the absence of blood flow, we therefore injected *tnnt2a*-MO alone or in combination with *gmfg* mRNA into zebrafish embryos and performed WISH analysis. Compared to their control siblings, embryos injected with *tnnt2a*-MO had a marked reduction in the number of *runx1*/*cmyb* + HSPCs along the DA at 36 hpf and 48 hpf, as demonstrated previously [43]. However, this effect was mitigated by the enforced expression of *gmfg* (Fig. 5c–f). Correspondingly, parallel results were obtained by utilizing *cd41*:eGFP transgenic embryos in which *gmfg* overexpression significantly increased the number and percentage of *cd41* + cells in *tnnt2a*-MO embryos at 80 hpf, as assessed by confocal microscopy (Fig. 5g, h) and flow cytometry (Fig. 5i). The role of *gmfg* in HSPC development and the requirement of blood flow for *gmfg* expression in HSPCs and hematopoietic tissues suggest that *gmfg* signaling is a part of the blood flow-mediated regulatory mechanism underlying HSPC development.

### *gmfg* regulates Yap activity

**Fig. 6:**
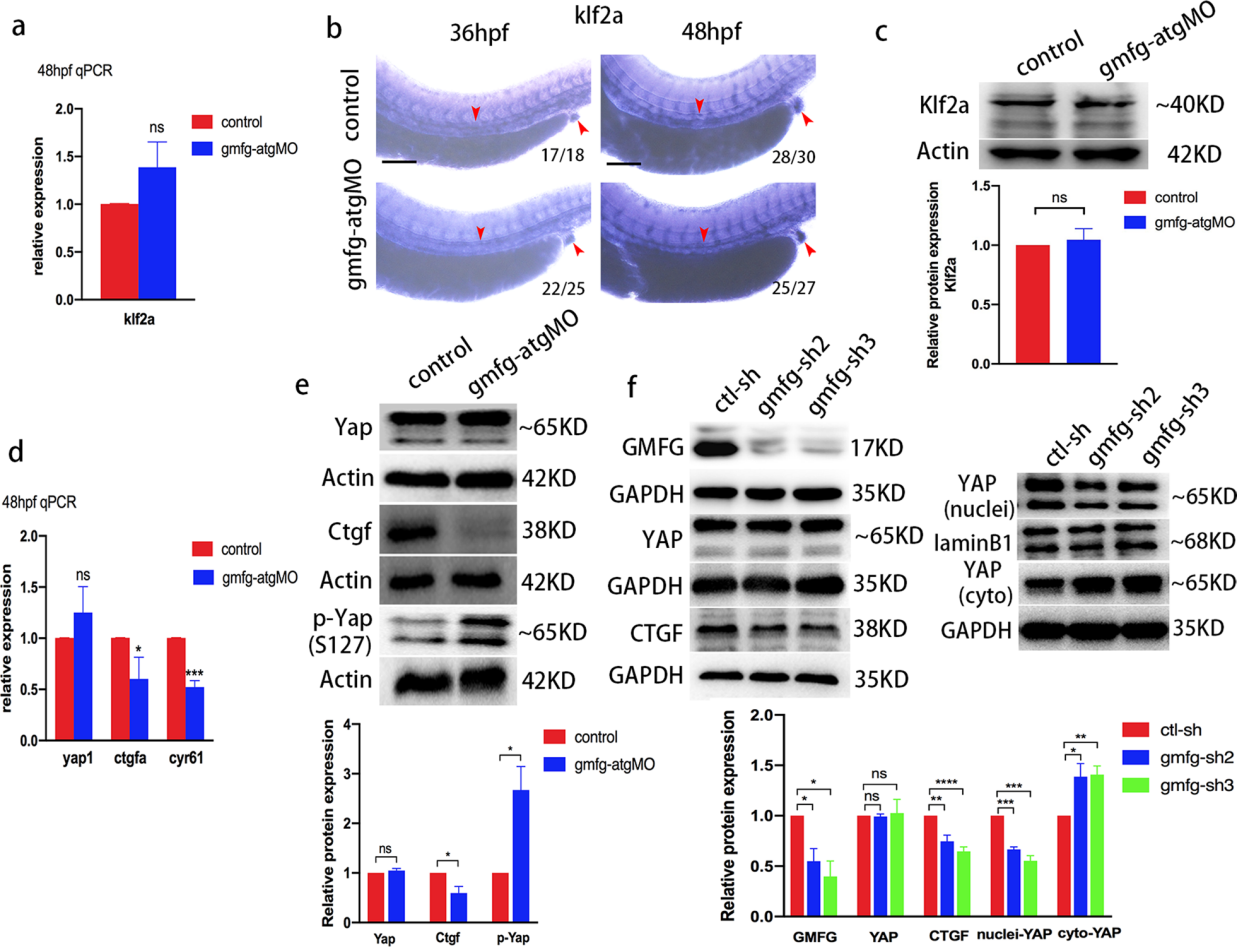
*gmfg* regulates Yap activity. **a** Quantitative RT-PCR analysis of *klf2a* expression in FACS-sorted HE of control and *gmfg*-atgMO embryos at 48 hpf. Bars represent mean ± SD (*n* = 3). ns, not significant. **b** WISH analysis of *klf2a* expression in the trunk and the cloaca (red arrowheads) of control and *gmfg*-atgMO embryos at 36 and 48 hpf, respectively. Numbers at the lower right corner of the picture represent embryos with displayed phenotype/whole embryos. Scale bars, 100 µm. **c** Western blotting showing the protein level of Klf2a in the dissected trunk and tail of control and *gmfg*-atgMO embryos at 54 hpf. Representative blot is shown in the figure (Full-length blots are presented in Additional file 2: Fig. S4). Data represent mean ± SEM intensity of indicated blots (*n* = 4). ns, not significant. **d** Quantitative RT-PCR analysis of *yap1*, *ctgfa* and *cyr61* expression in FACS-sorted HE of control and *gmfg*-atgMO embryos at 48 hpf. Bars represent mean ± SD (*n* = 3). ns, not significant; **p < 0.05, ***p < 0.001. **e** Western blotting showing the protein level of Yap, Ctgf and p-Yap (S127) in the dissected trunk and tail of control and *gmfg*-atgMO embryos at 48 hpf. Representative blot is shown in the figure (Full-length blots are presented in Additional file 2: Figs. S5–S7). Data represent mean ± SEM intensity of indicated blots (*n* = 3). ns, not significant; *p < 0.05. **f** Western blotting showing the protein level of GMFG, total YAP, CTGF, nuclear and cytoplasmic YAP in HUVEC treated with ctl-sh, *gmfg*-sh2 and *gmfg*-sh3. Representative blot is shown in the figure (Full-length blots are presented in Additional file 2: Figs. S8–S12). Data represent mean ± SEM intensity of indicated blots (*n* ≥ 3). ns, not significant; *p < 0.05; **p < 0.01; ***p < 0.001, ****p < 0.0001

When pulsatile blood flow goes through a vessel, it generates both shear stress and circumferential strain [44, 45]. Recent studies have suggested that these two different components of hemodynamic forces regulate HSPC development via distinct and separable mechanisms [10, 43]. Thereinto, kruppel-like transcription factor 2 (Klf2) was revealed to be an important mechanical mediator sensitive to fluid shear stress [43, 46] while YAP has been recently identified as a circumferential strain-induced regulator of HSPC formation in zebrafish [10]. To explore how *gmfg* relays signals from blood flow to HSPCs, we naturally tended to analyze the effects of *gmfg* deficiency on these two well-known flow-responsive factors. qPCR analysis of *klf2a* in FACS-sorted HE at 48 hpf demonstrated that the mRNA level of *klf2a* in *gmfg* morphants was comparable to that in controls (Fig. 6a). *klf2a* expression assayed by WISH, specifically in the axial vessels also did not differ significantly between control and *gmfg* morphants at 36 hpf and 48 hpf (Fig. 6b). Moreover, western blot analysis of the protein level of Klf2a from the dissected trunk and tail at 54 hpf further supported the validity of WISH and qPCR results (Fig. 6c). These data suggest, in 3 independent settings, that *gmfg* deficiency has little effect on *klf2a* expression, excluding the possibility of *klf2a* as a *gmfg*-target gene. Then, we aimed to characterize the impact of *gmfg* deficiency on Yap. As shown in Fig. 6d, loss of *gmfg* had little effect on the mRNA level of *yap1* (zebrafish YAP gene) but significantly reduced the expression of two well-known Yap target genes, *ctgfa* and *cyr61*, in FACS-sorted HE at 48 hpf, suggestive of repressed Yap signaling. This observation was further supported by western blot analysis of dissected trunk and tail at 48 hpf, in which Yap expression was comparable between control and *gmfg* morphants but Ctgf expression was remarkably downregulated in *gmfg* morphants (Fig. 6e, upper and medium panel). Since phosphorylation of YAP on serine 127 (S127) generally underlies YAP inactivation and cytoplasmic sequestration [47], we further analyzed the phosphorylation of Yap (S127) in zebrafish. As anticipated, an increased level of p-Yap (S127) was observed in embryos injected with *gmfg*-atgMO (Fig. 6e, lower panel), suggesting that *gmfg* deficiency facilitated cytoplasmic sequestration of YAP.

In order to further verify the relationship between *gmfg* and Yap, both of which are downstream effectors of blood flow, we extended our analysis in vitro by utilizing HUVEC cell line. The reason why we selected HUVEC lies in the generation of embryonic HSCs from HE, and to some extent, HE is considered to be a special subtype of endothelium. Besides, as endothelium lining the surface of blood vessels are naturally exposed to blood flow, their mechanotransduction process elicited by flow is relatively well elucidated [48]. Previous reports have demonstrated that in endothelium blood flow promotes Yap nuclear localization via actin cytoskeleton reorganization [49]. Combined with the fundamental role of *gmfg* in actin network remodeling [13], the choice of HUVEC seems to be both reasonable and convenient. Firstly, we found that the protein level of GMFG was significantly decreased in *gmfg*-sh2 and *gmfg*-sh3 HUVEC, once again verifying the efficacy of these two shRNAs (Fig. 6f, left upper panel). Then, further western blot results showed that knockdown of *gmf*g in HUVEC had no effect on YAP expression but significantly attenuated the protein level of CTGF (Fig. 6f, left medium and lower panel), corroborating the results observed in zebrafish. In addition, a significantly reduced YAP level in the nucleus but an increased YAP level in the cytoplasm were observed in *gmfg* knockdown HUVEC (Fig. 6f, right). These data altogether indicated that loss of *gmfg* prevents the shuttling of endogenous YAP from cytoplasm to nucleus and thus attenuated YAP signaling. Collectively, we conclude that *gmfg* mediates blood flow-induced HSPC development via regulation of Yap activity in zebrafish.

### *gmfg* acts upstream of Notch during HSPC initiation

HSPC programming has been proposed to initiate (24 hpf) normally but not properly maintain (from 36 hpf onward) in the absence of circulation [43]. However, findings reported here showed that both the early initiation and the later maintenance of HSPCs (24 hpf and onward) were impeded in a *gmfg*-deficient background. Therefore, blood flow alone cannot explain all the cell fate changes in HSPCs and there must be another mechanism responsible for the defects in HSPC initiation. To resolve this issue, we first assume that *gmfg* potentially crosstalk with developmental pathways known to be essential for the establishment of HSPC fate, including Notch [50], BMP [51], Hedgehog [51, 52] and cAMP signaling [53]. Accordingly, we screened these pathways using an unambiguous WISH rescue assay for expression of *runx1/cmyb*—that is, application of specific inhibitors or agonists along with injection of *gmfg* mRNA or *gmfg*-atgMO—and analyzed their corresponding rescue of inhibitor- or loss of *gmfg*-induced HSPC defects. However, *gmfg* overexpression could not significantly rescue the negative effects of inhibition of BMP, Hedghog and Notch signaling on the number of HSPCs (Additional file 1: Fig. S7a, c, d, f), and activation of cAMP signaling also could not restore the HSPC loss caused by *gmfg* deficiency (Additional file 1: Fig. S7b, e), indicating that pathway crosstalk may not be the main mechanism, and that *gmfg* may directly regulate HSPC initiation through its downstream signaling events.

**Fig. 7:**
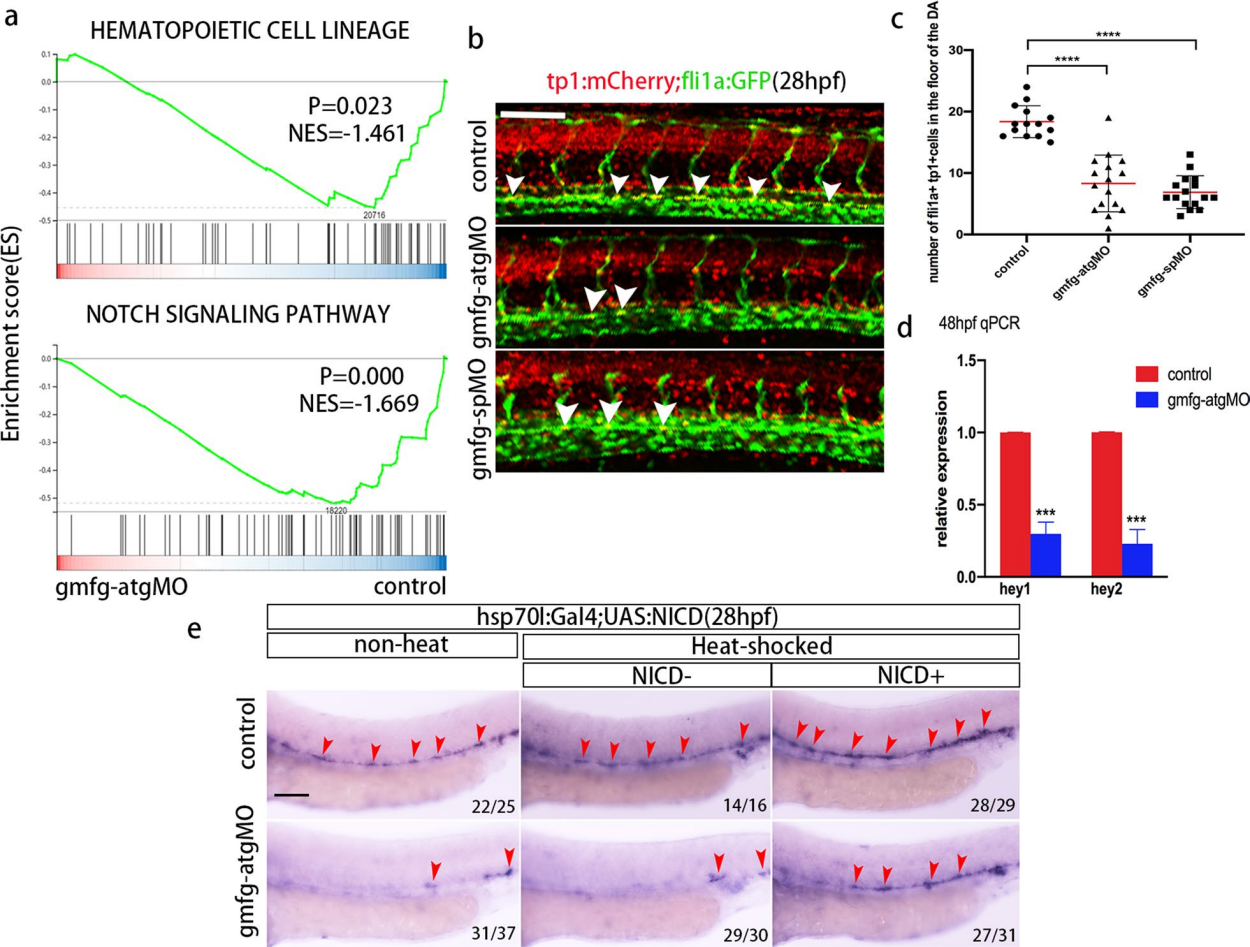
*gmfg* acts upstream of Notch during HSPC initiation. **a** GSEA profiles depicting the enriched processes in “hematopoietic cell lineage” and “Notch signaling pathway”. **b** *tp1*:mCherry;*fli1a*:eGFP double transgenic embryos injected with control MO, *gmfg*-atgMO, and *gmfg*-spMO were visualized at 28 hpf. White arrowheads indicate cells in the DA with active Notch signaling. **c** Enumeration of *tp1* + *fli1a* + Notch-active ECs from (b). Bars represent mean ± SD of control (*n* = 14), *gmfg*-atgMO (*n* = 16), and *gmfg*-spMO (*n* = 16) embryos. ****p < 0.0001. **d** Quantitative RT-PCR results of Notch target genes *hey1* and *hey2* in FACS-sorted HE of control and *gmfg*-atgMO embryos at 48 hpf. Bars represent mean ± SD (*n* = 3). ***p < 0.001. **e** *hsp70l*:Gal4;UAS:NICD embryos injected with control MO and *gmfg*-atgMO were heat shocked at 12–14 hpf and WISH for *runx1* was performed at 28 hpf. Arrowheads denote *runx1* + HSPCs along the DA. Numbers at the lower right corner of the picture represent embryos with displayed phenotype/whole embryos. All scale bars, 100 µm

To more specifically explore the underlying mechanism by which *gmfg* regulates HSPC initiation, we performed RNA-sequencing (RNA-Seq) with the dissected trunk and tail from 26 hpf *gmfg* morphants and their control siblings. However, according to GO enrichment analysis and Kyoto Encyclopedia of Genes and Genomes (KEGG) pathway enrichment analysis, DEGs were not enriched in hematopoietic-related pathway, possibly because of the scarcity of hematopoietic cells in the dissected trunk and tail tissue. Instead, gene set enrichment analysis (GSEA) profiles revealed that hematopoietic cell lineage-related genes were significantly enriched in the control group as compared to *gmfg* morphant group (Fig. 7a, upper panel), consistent with the impairment of hematopoiesis upon *gmfg* deficiency. In addition, among the transcriptional programs downregulated in *gmfg* morphants, Notch signaling pathway was especially attractive because of the enrichment of Notch signaling pathway-related genes in the control group (Fig. 7a, lower panel) and its evolutionarily conserved role in specifying and regulating definitive HSPCs [50, 54]. The inactivation of Notch signaling induced by *gmfg* deletion was first validated by utilizing individual *tp1*:GFP transgenic lines whose fluorescence intensity of GFP reflects Notch activity [55]. In *gmfg* morphants, GFP expression along the floor of the DA was weak and discontinuous at 30 hpf (Additional file 1: Fig. S8a), indicative of attenuated Notch activity. Flow cytometry analysis also revealed an obvious reduction in the percentage of *tp1* + Notch-activated cells in whole embryos upon *gmfg* deficiency (Additional file 1: Fig. S8b). To further authenticate the results, the number of *tp1* + *fli1a* + double positive cells within the aortic floor was quantified in *fli1a*:eGFP;*tp1*:mCherry double-transgenic embryos at 28 hpf, and a significantly reduced number of Notch-active ECs was observed in *gmfg* morphants (Fig. 7b, c). Furthermore, qPCR analysis showed that the expression of the Notch target genes *hey1* and *hey2* was decreased in FACS-sorted HE at 48 hpf upon *gmfg* deficiency (Fig. 7d). These lines of evidence, in combination with above findings that the enforced expression of *gmfg* could not rescue the depletion of *runx1*/*cmyb* + HSPCs in the Notch-specific inhibitor DAPT-treated embryos (Additional file 1: Fig. S7c, f), we believe that Notch signaling is a downstream pathway of *gmfg*.

If Notch signaling is indeed required downstream of *gmfg* for HSPC initiation, then the induction of Notch intracellular domain (NICD) should rescue the HSPC loss observed in *gmfg* morphants. Therefore, we performed timing-specific Notch signaling rescue experiment. To provide temporal control of NICD induction, we utilized inducible *hsp70l*:Gal4;UAS:NICD double-transgenic embryos, which express NICD under the control of the inducible Gal4 system. WISH results showed that heat-shock induced overexpression of NICD at 12–14 hpf could partially restore the decrease of *runx1* expression at 28 hpf in *gmfg*-deficient embryos (Fig. 7e). Altogether, these results indicated that the impaired Notch signaling in *gmfg* morphants led to the failure of HSPC to be initiated and that Notch acts downstream of *gmfg* during HSPC initiation.

## Discussion

A previous study in mice reported that *gmfg* is highly expressed in the DA where embryonic HSCs emerge [15], implying a potential regulatory role of *gmfg* in definitive hematopoiesis but the detailed mechanisms remain unclear. Here, by identifying *gmfg* as an important regulator of zebrafish HSPC development, we define a novel conceptual framework for analysis of *gmfg* biology. Given that *gmfg* is highly conserved among vertebrates, with 76% amino acid sequence identity between zebrafish and human and 94% identity between mice and human [13], it is logical to explore the function of *gmfg* in zebrafish to predict its role in human. In addition, as a brain-specific *gmf* isoform, *gmfb* is preferentially expressed in astrocytes and some neuronal populations, playing an essential role in the development and differentiation of neurons and glia instead of hematopoiesis [13, 56]. BIOGPS database also confirmed its nervous system expression preference (Additional file 1: Fig. S9), which may be part of the reason why both *gmf* isoforms have a common ADF-H domain but knock down of *gmfb* did not affect HSPC development. Furthermore, evidence suggests that *gmf* activities are regulated in the manner of post-translational modification [57]. Structurally, except for several conserved phosphorylation sites shared by *gmfg* and *gmfb*, *gmfg* has two additional PKC and CKII kinase consensus sites that are not found in *gmfb *[58]. This distinguishable structural difference might result in the distinct or only partially overlapping roles of *gmfg* and *gmfb*, though it remains to be investigated into how phosphorylation at each of these sites impacts their activities and cellular functions.

In this study, we demonstrate that loss of *gmfg* leads to the blockage of EHT and consequently the depletion of the emerging HE (Fig. 2g, h). Traditionally, EHT is reminiscent of the more well-established epithelial-to-mesenchymal transition (EMT), both of which are cell fate change programs giving rise to live wandering cells endowed with a novel potential [59, 60]. In some studies EHT was regarded as a partial EMT [61] as HSPCs acquire only partial EMT identity after EHT, followed by a transition to a more mesenchymal status with acquisition of migratory and invasive traits required for colonization of sequential hematopoietic sites [62]. During tumor progression, tumor cells undergo invasion and metastasis through EMT [63]. Interestingly, *gmfg* promoted EMT of tumor cells [64], and it was revealed to be highly associated with tumor prognosis including leukemia [23, 64–66]. Moreover, externally applied shear stress is able to induce an EMT-like process termed endothelial-to-mesenchymal transition (EndMT) at a low value compatible to the magnitude experienced by the developing embryo during EHT [67]. These lines of evidence logically supported the function of *gmfg* in EHT and the conserved role of *gmfg* in these two similar cell transitions. Therefore, it is reasonable to consider whether the findings reported in our study may also apply in other types of tumor stem cells. Given that in certain studies *gmfg* was considered as a potential predictive factor in the development of cancer [23, 68], the impact of biomechanical microenvironment should be taken into account in the treatment of diseases related to *gmfg*.

There is no doubt that the mechanism of *gmfg* in definitive hematopoiesis appears to be complicated. In this paper, the regulation of embryonic HSPC development by *gmfg* can be divided into two parts: HSPC initiation before the action of circulation and HSPC maintenance under the control of blood flow. In addition to its involvement in blood flow-dependent HSPC maintenance via regulation of Yap, our results also suggest that *gmfg* exerts its effects on HSPC initiation through Notch. However, the relationship between YAP and Notch signaling cascades in the *gmfg*-regulatory context remains to be investigated. Two main interaction modalities between them have been revealed in diverse cellular contexts: YAP control of Notch ligands or receptors, and joint regulation of common target genes by YAP and NICD [69]. It seems that in our study, the latter may be true, in which NICD and Yap are independent downstream effectors of *gmfg*, because these two pathways act in sequential order and *gmfg*-Notch signaling precedes the blood flow-*gmfg*-Yap axis. However, previous studies reported that in blood flow-deficient embryos, the expression of *ephrinb2a*, a Notch target gene, albeit remained unaffected at 24 hpf, but was dramatically downregulated at 36 hpf [43]. Another study in mice also demonstrated that loss of blood flow resulted in suppression of Notch signaling in paraaortic splanchnopleura (PSp)-derived hematopoietic precursors, while application of wall shear stress simulating blood flow to mouse AGM cells cultured in vitro was sufficient to activate multiple hematopoietic regulatory signaling pathways, including Notch [70]. These experimental evidences suggest that blood flow is capable of activating the Notch pathway. Here, we also observed reduced expression of *ephrinb2a* and *dlc* at 28 hpf and the persistent down-regulation of Notch signaling from 28 to 48 hpf in *gmfg*-deficient embryos, which implies the temporal coincidence of the *gmfg*-Notch and blood flow-*gmfg*-Yap pathways, however whether interactions between them exist remains unknown.

## Conclusions

In summary, we present evidence for the first time that *gmfg*, a regulator in actin dynamics, plays a key role in the establishment of embryonic HSPC fate. We found that *gmfg* not only controls HSPC initiation by activating endogenous Notch signaling, but also participates in HSPC maintenance by mediating microenvironmental blood flow inputs. *gmfg* represents a multifunctional factor responsible for the broad support of HSPCs, and thus *gmfg* signals should now be included in the list of known signaling inputs required for HSPC development. Manipulation of molecular pathways related to *gmfg* may provide novel insights into the generation of bona fide HSPCs in vitro.

## Supplementary Information

**Additional file 1.** Supplementary figures and tables.

**Additional file 2.** Corresponding original, unprocessed scans of blots. The original, unprocessed images of blots shown in Figure 2b, Figure 5b, Figure 6c, Figure 6e, Figure 6f, Supplementary Figure 1a, Supplementary Figure 1b, Supplementary Figure 2b, and Supplementary Figure 3 are listed. ctl denotes control group, aMO denotes *gmfg*-atgMO group, sMO denotes *gmfg*-spMO group, tMO denotes *tnnt2a*-MO group, sh2 denotes *gmfg*-sh2 group and sh3 denotes *gmfg*-sh3 group.

**Additional file 3.** *lcr*:eGFP transgenic embryos were injected with control MO and visualized at 48 hpf.

**Additional file 4.** *lcr*:eGFP transgenic embryos were injected with tnnt2a MO and visualized at 48 hpf.

## Data Availability

The raw sequence reads of RNA-seq data presented in the paper were deposited at Sequence Read Archive (SRA) under the accession number PRJNA884084. The Submission ID is SUB12099273. All data generated or analyzed during this study are included in this published article and the Additional files.

## Abbreviations

HSPCs: Hematopoietic stem and progenitor cells
HSCs: Hematopoietic stem cells
PSCs: Pluripotent stem cells
HE: Hemogenic endothelium
EHT: Endothelial-to-hematopoietic transition
AGM: Aorta-gonad-mesonephros
CHT: Caudal hematopoietic tissue
WISH: Whole-mount in situ hybridization
MO: Morpholino oligonucleotide
EPCR: Endothelial protein C receptor
GSEA: Gene set enrichment analysis
NICD: Notch intracellular domain
PLM: Posterior lateral mesoderm
EMT: Epithelial-to-mesenchymal transition
