# Supplementary material for: Glia maturation factor-γ is required for initiation and maintenance of hematopoietic stem and progenitor cells
Source: Stem Cell Res Ther. 2023 Apr 30;14:117. doi: 10.1186/s13287-023-03328-1 (PMC10150485; doi:10.1186/s13287-023-03328-1)
Supplement: Supplementary file 1 — Additional file 1. Supplementary figures and tables. [file 13287_2023_3328_MOESM1_ESM.docx]

Additional File 1

Glia maturation factor-γ is required for initiation and maintenance of hematopoietic stem and progenitor cells

**By**

Honghu Li^1,2,3,4,*^, Qian Luo^1,2,3,4,*^, Shuyang Cai^1,2,3,4,*^, Ruxiu Tie^1,2,3,4^,Ye Meng^1,2,3,4^, Wei Shan^1,2,3,4^, Yulin Xu^1,2,3,4^, Xiangjun Zeng^1,2,3,4^, Pengxu Qian^1.2.3.4.5.6🖂^, He Huang^1,2,3,4.5🖂^

*These authors contributed equally to this work: Honghu Li, Qian Luo, Shuyang Cai.

Supplementary Figures

Figure S1


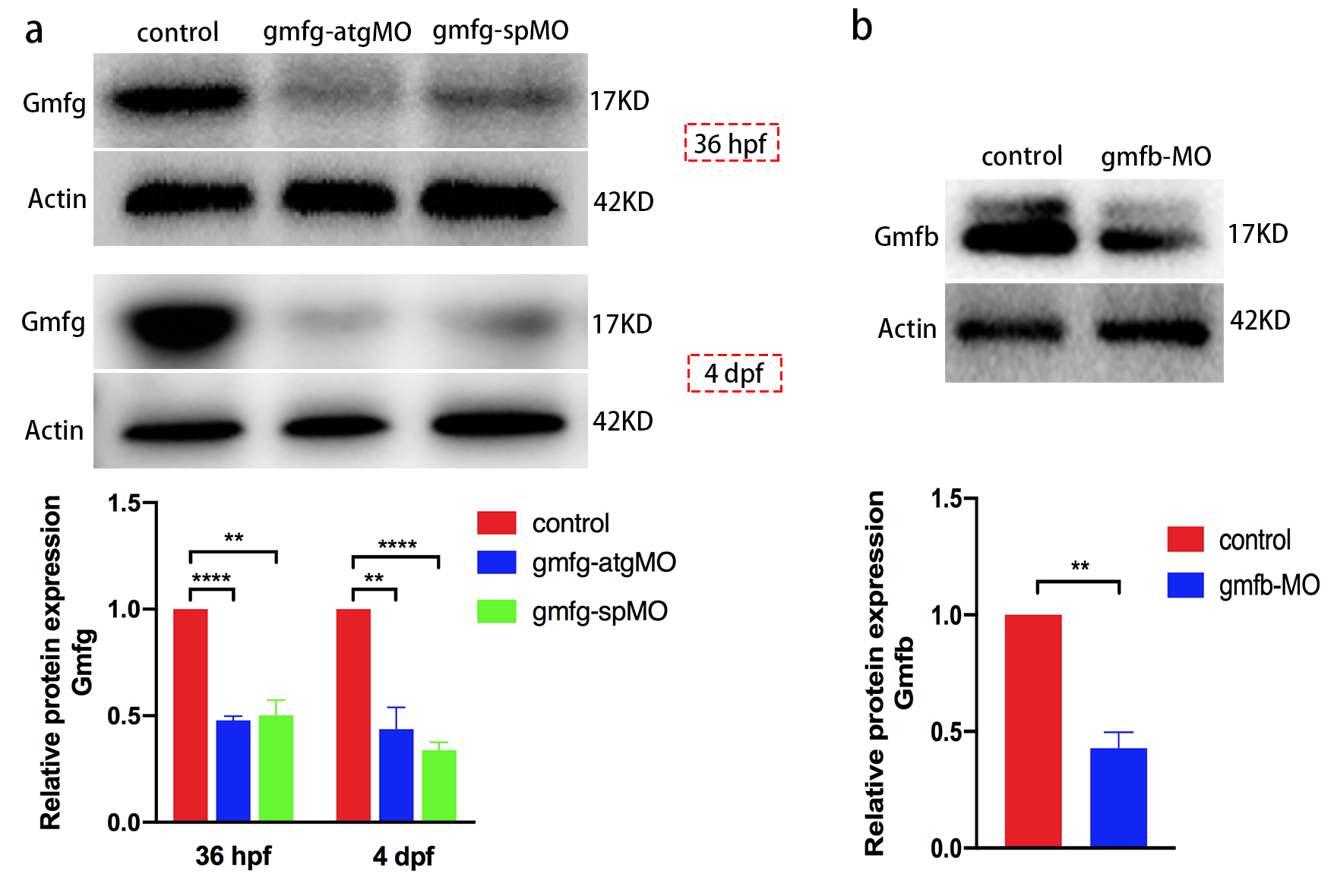


Figure S2


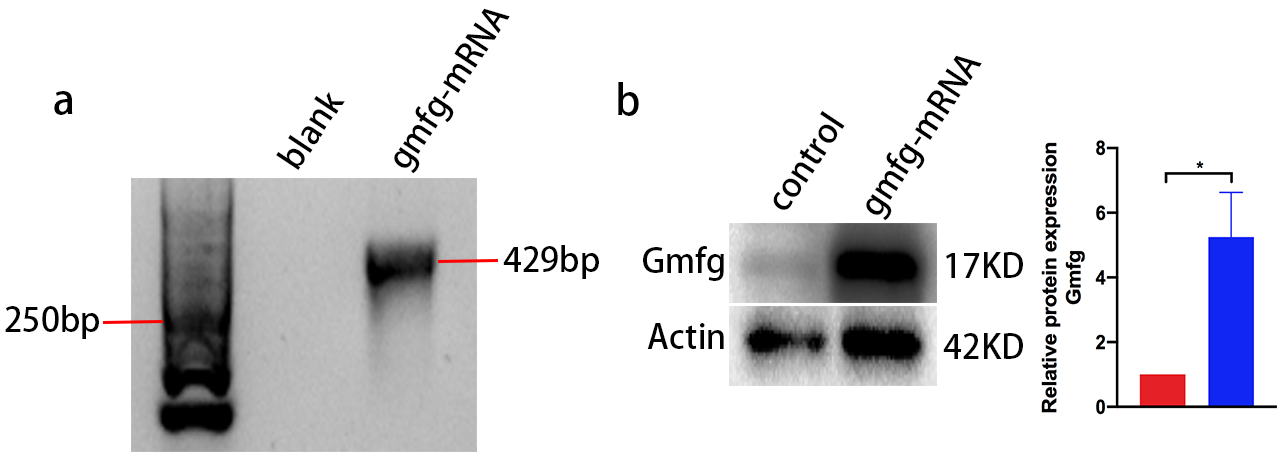


Figure S3


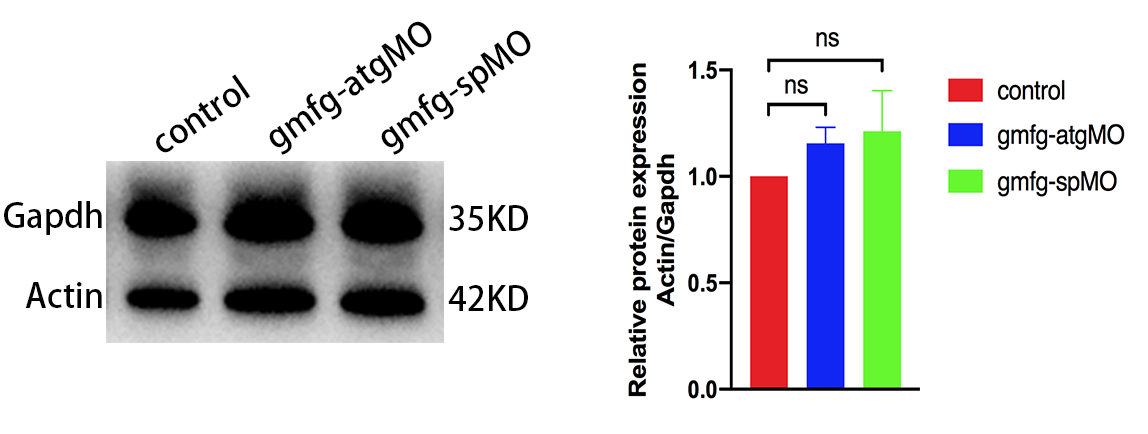


Figure S4


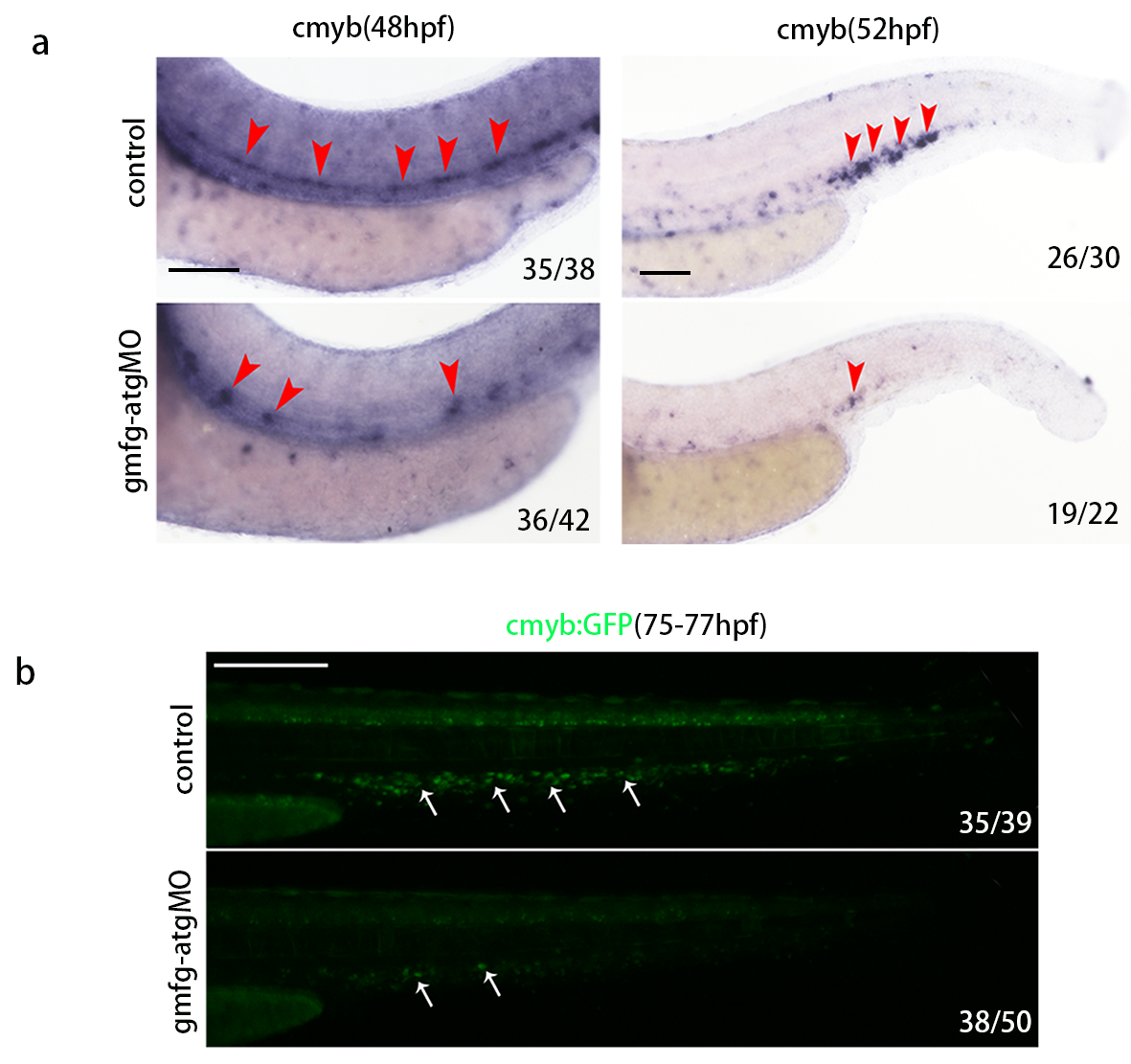


Figure S5


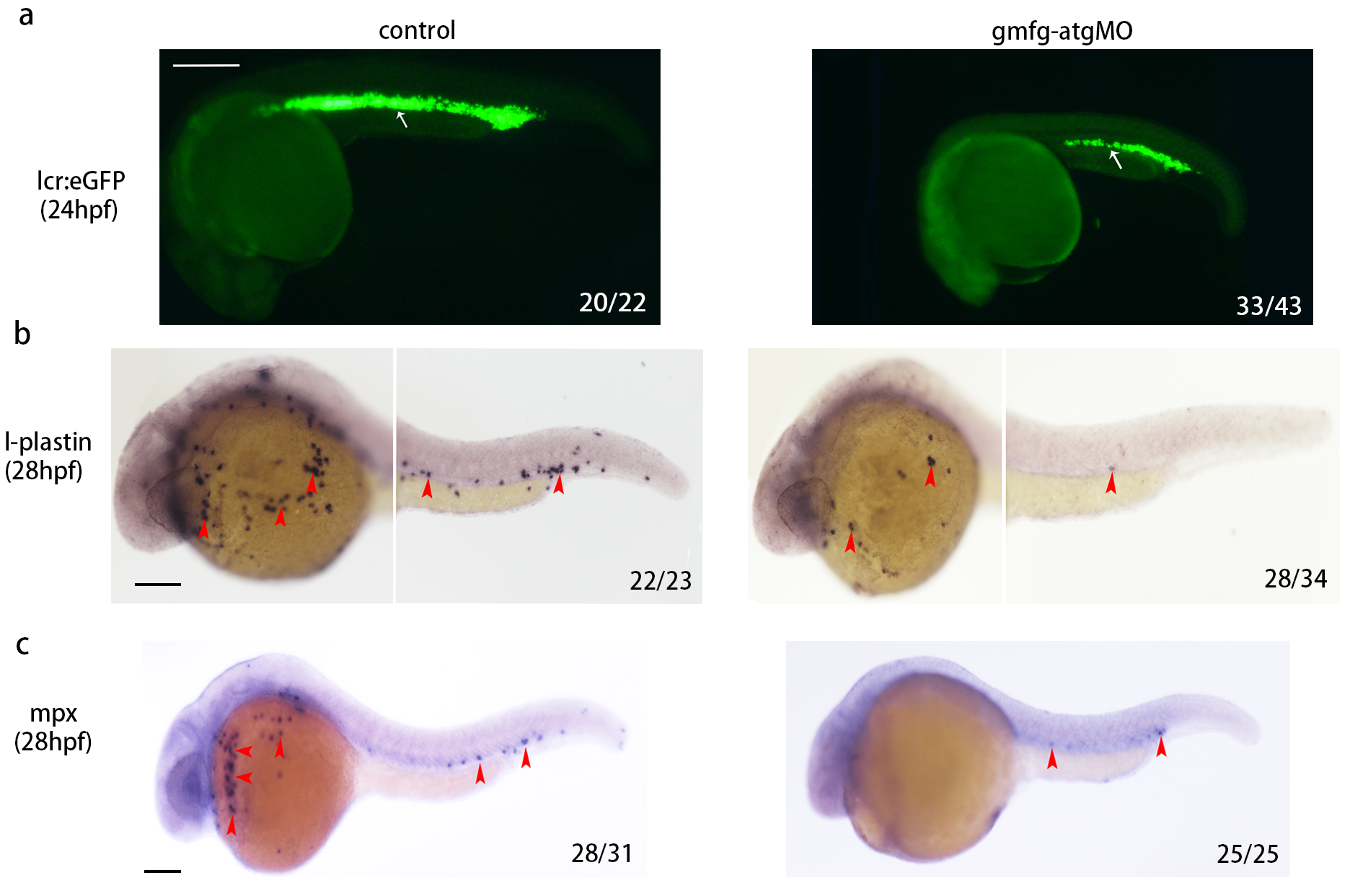


Figure S6


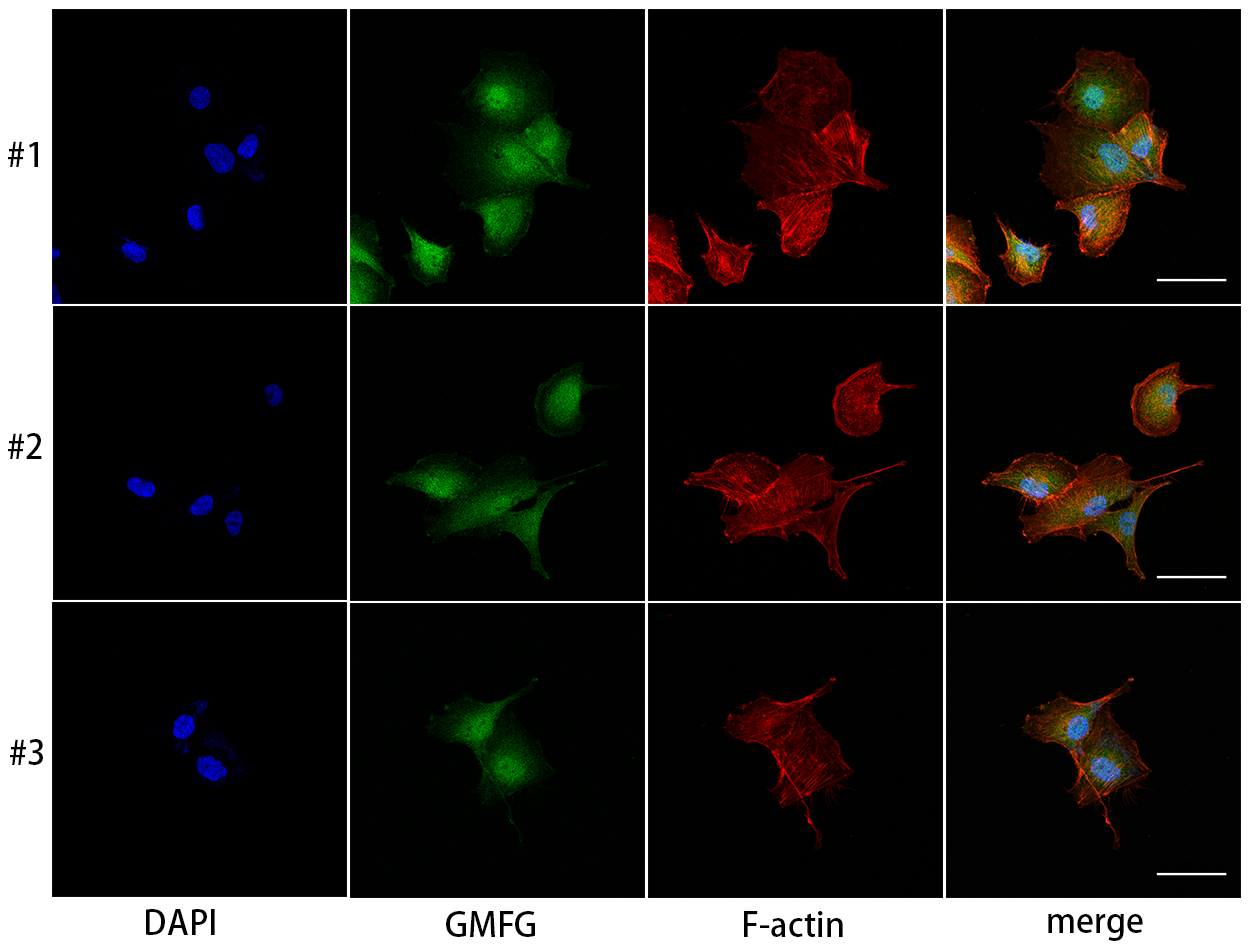


Figure S7


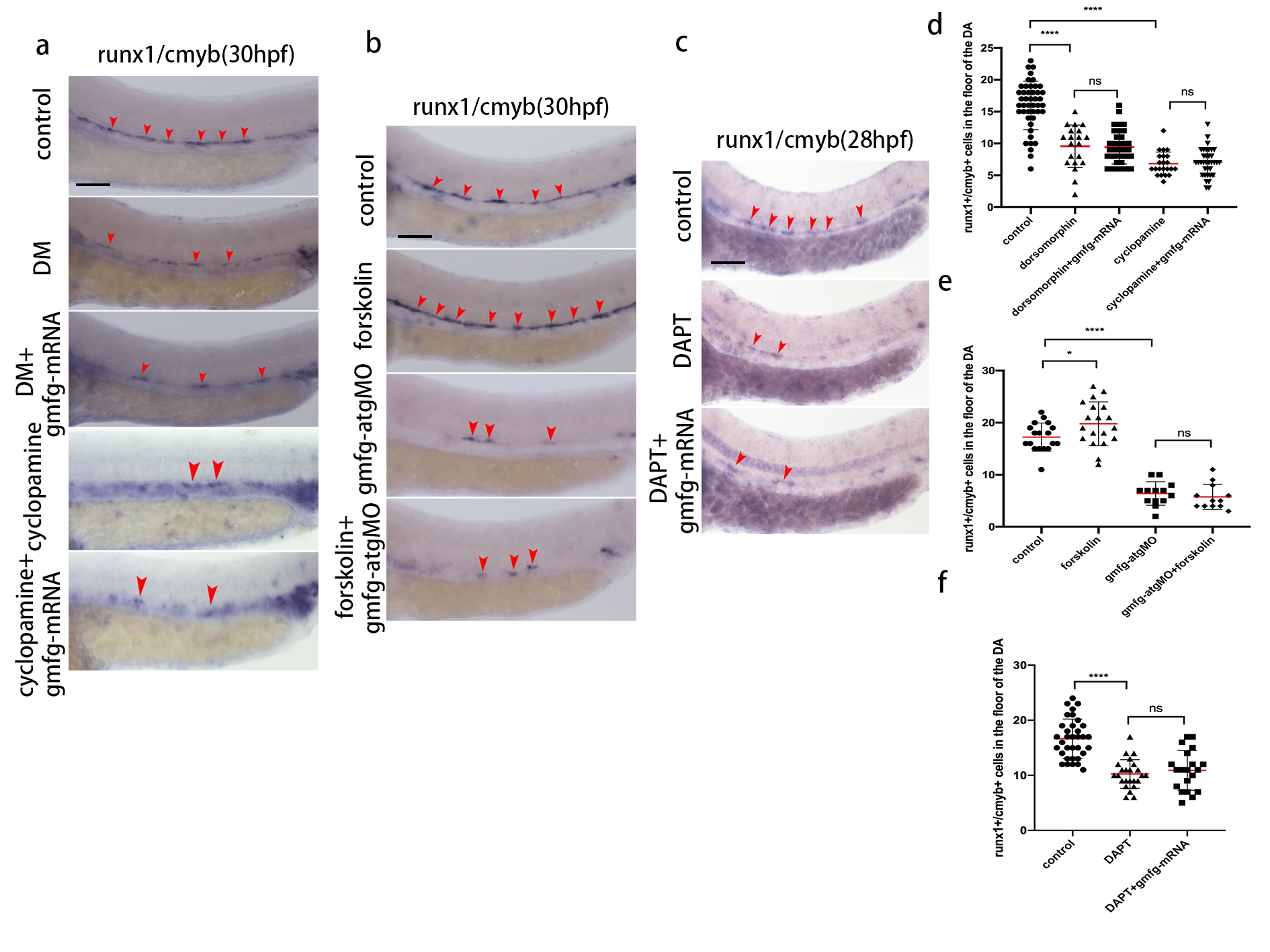


Figure S8


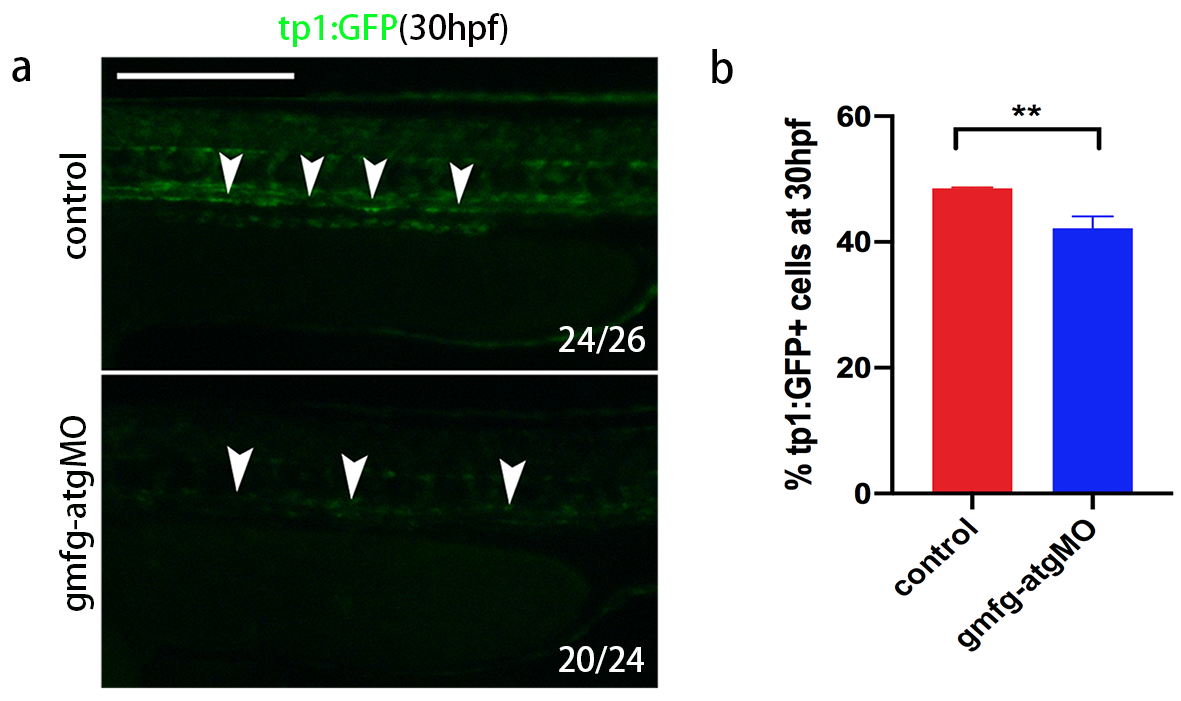


Figure S9

**
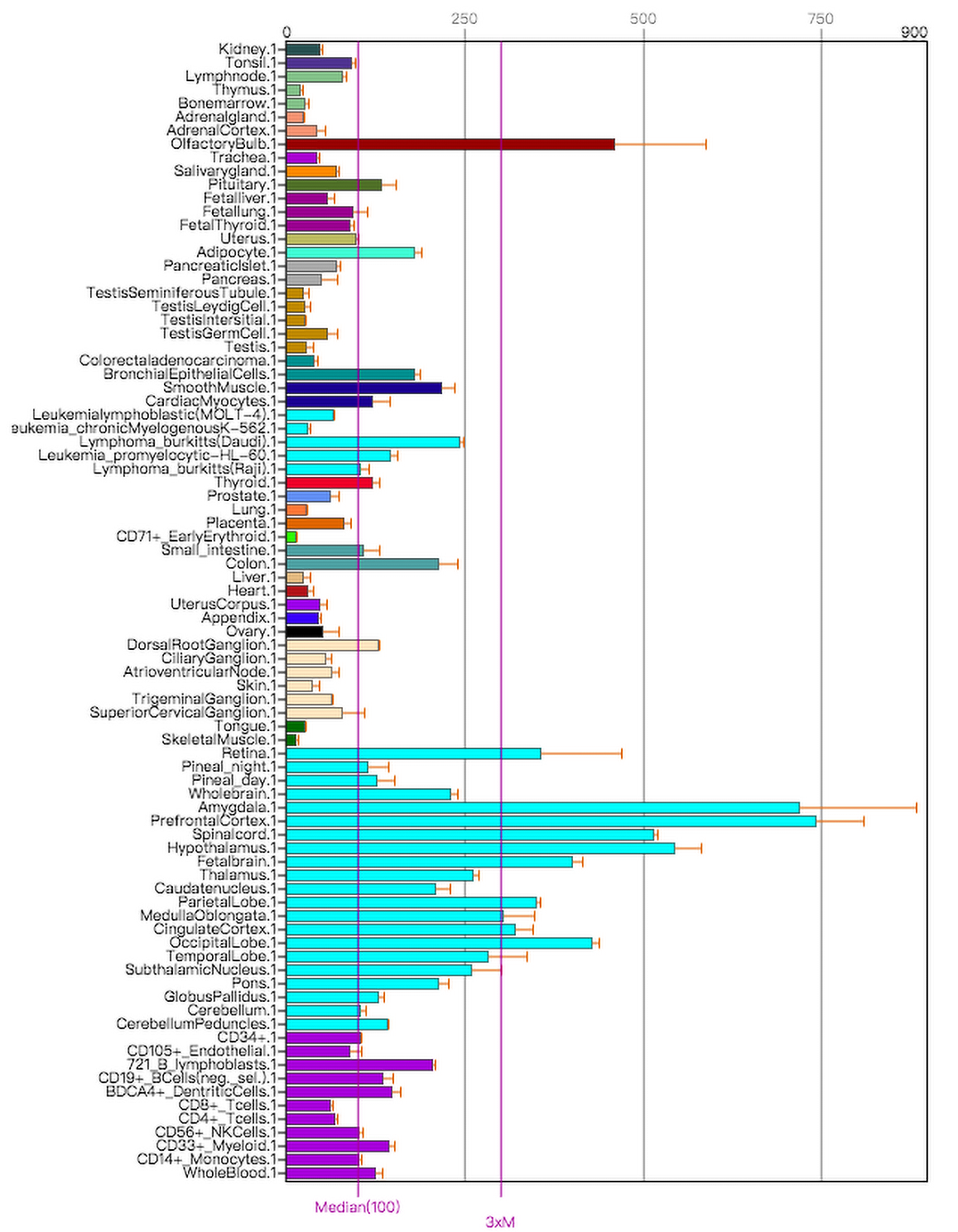
**

**Supplementary Figure Legend**

**Supplementary Figure. 1 Validation of the efficiency of *gmfg* and *gmfb* MOs.** Western blotting showing the protein level of Gmfg at 36 hpf and 4 dpf (a), and Gmfb at 48 hpf (b) following indicated MO injection. Representative blots are shown in the figures (Full-length blots are presented in Additional file 2: Fig. S13-S15). Data represent mean ± SEM intensity of indicated blots (n=3). **p < 0.01; ****p < 0.0001.

**Supplementary Figure. 2 Validation of the specificity of *gmfg* mRNA. a** Agar gel electrophoresis validated the *gmfg* mRNA band is correct in size (429bp). **b** Western blotting showing the protein level of Gmfg in untreated embryos (control) and embryos injected with 100pg *gmfg* mRNA at 48 hpf. Representative blot is shown in the figure (Full-length blots are presented in Additional file 2: Fig. S16). Data represent mean ± SEM intensity of indicated blots. *p < 0.05.

**Supplementary Figure. 3 Validation of the expression stability of Actin.** Western blotting showing the protein level of Actin compared with Gapdh at 48 hpf following knockdown of *gmfg*. Representative blot is shown in the figure (Full-length blots are presented in Additional file 2: Fig. S17). Data represent mean ± SEM intensity of indicated blots (n=3). ns, not significant.

**Supplementary Figure. 4 Time course analysis of *cmyb* expression reveals HSPC defects were specific to *gmfg*. a** WISH analysis showing *cmyb* expression (red arrowheads) in the DA (left) and the CHT (right) of control and *gmfg* morphants at 48 and 52 hpf, respectively. Scale bars, 100 μm. **b** *cmyb*:GFP transgenic embryos were injected with control MO and *gmfg*-atgMO and visualized at 75-77 hpf. White arrowheads indicate *cmyb*+ HSPCs in the CHT. Scale bar, 250 μm. Numbers at the lower right corner of the picture represent embryos with displayed phenotype/whole embryos.

**Supplementary Figure. 5 Primitive hematopoiesis is impaired in *gmfg* morphants. a** *lcr*:eGFP transgenic embryos were injected with control MO and *gmfg*-atgMO and visualized at 24 hpf, white arrowheads indicate *lcr*+ primitive erythrocytes in the intermediate cell mass (ICM). Scale bar, 250 μm. **b and c** WISH analysis showing the expression of *l-plastin* (b; red arrowheads) and *mpx* (c; red arrowheads) in the anterior lateral mesoderm (ALM) and the posterior lateral mesoderm (PLM) of control and *gmfg* morphants at 28 hpf. Anterior is to the left and dorsal is right. Numbers at the lower right corner of the picture represent embryos with displayed phenotype/whole embryos. Scale bars, 100 μm.

**Supplementary Figure. 6 Immunostaining of F-actin and GMFG in HUVEC.** Colocalization of F-actin and GMFG in the forward periphery of cells and cytoplasm was observed in HUVEC. Scale bars, 50 μm. Cells from 3 fields are showed. The fluorescent image resolution is 1024×1024 pixels.

**Supplementary Figure. 7 Zebrafish embryos were exposed to indicated chemicals from 12 hpf until fixation and subjected to WISH for *runx1/cmyb*. a** Representative images showing *runx1/cmyb* expression (red arrowheads) in the DA of control, dorsomorphin (DM), DM+*gmfg*-mRNA, cyclopamine, and cyclopamine+*gmfg*-mRNA embryos at 30 hpf. **b** Representative images showing *runx1/cmyb* expression (red arrowheads) in the DA of control, forskolin, *gmfg*-atgMO, and forskolin+*gmfg*-atgMO embryos at 30 hpf. **c** Representative images showing *runx1/cmyb* expression (red arrowheads) in the DA of control, DAPT, and DAPT+*gmfg*-mRNA embryos at 28 hpf. **d-f** Enumeration of *runx1*+/*cmyb*+ HSPCs from (a-c). Bars represent mean ± SD, n=45, 20, 34, 22, 35 (a), n=18, 19, 13, 12 (b), n=34, 24, 20 (c). ns, not significant; *p < 0.05, ****p < 0.0001. Scale bars, 100 μm.

**Supplementary Figure. 8 *gmfg* deletion results in the inactivation of Notch signaling. a** *tp1*:GFP transgenic embryos were injected with control MO and *gmfg*-atgMO and visualized at 30 hpf. White arrowheads indicate Notch activity along the DA. Scale bar, 250μm. Numbers represent embryos with displayed phenotype/whole embryos. **b** FACS analysis showing the percentage of *tp1*+ cells in whole embryos of control and *gmfg*-atgMO groups at 30 hpf (n = 3). **p < 0.01.

**Supplementary Figure. 9 *gmfb* expression in human tissues.** The abscissa represents different human tissues, the ordinate represents gene expression level, and the histogram represents the expression level of *gmfb* in specific human tissue.

**Supplementary Tables**

**Supplementary Table1 Primers for genotyping of transgenic lines.**

| Name | Sequence (from 5’ to 3’) |
| --- | --- |
| P53-WT | F: GATAGCCTAGTGCGAGCACACTCTT  R: AGCTGCATGGGGGGGAT |
| P53-mutant | F: AGCTGCATGGGGGGGAA  R: AGCTGCATGGGGGGGAT |
| hsp70l:Gal4 | F: CCGCTGACTAGGGCACAT  R: GACGGCATCTTTATTCACATTA |
| UAS:NICD | F: GAGAGCTTGGGCGACCTCA  R: CGTGCTGCCAGATGAAGTGC |

**Supplementary Table2 MOs used in this study.**

| MOs | Sequence (from 5’ to 3’) | reference |
| --- | --- | --- |
| gmfg-atgMO | CTCGACATCCTCAGTCTGTGTGTGT | [1] |
| gmfg-spMO | CAGACGACGACCAGCTCTTACCATC | This study |
| gmfb-MO | CACACACAACTAATGACTCACTCAT | [1] |
| tnnt2a-MO | TAGACACAGATGAACTCACAATTTC | [2] |
| Control-MO | standard MO provided by GeneTools | [3] |

**Supplementary Table3 qPCR and RT-PCR primers used in this study.**

| Gene Name | Sequence (from 5’ to 3’) | Species |
| --- | --- | --- |
| *gmfg* | F: ACAAGTACGTGCATGACGATG  R: GCCTGTTTTTACTCCCTGCATAC | human |
| *gmfb* | F: ATGTTGCCGAAGATTTAGTGGAA  R: CCACCAGGCGTTTATCCTTGT |  |
| *actin* | F: CATGTACGTTGCTATCCAGGC  R: CTCCTTAATGTCACGCACGAT |  |
| *klf2a* | F: ACCTATTGCTTGTAGCTGGTT  R: TAAAAGGCACTGTCGTGATG | zebrafish |
| *yap1* | F: ACATCATGAACCCAGCCTCAG  R: TGCTGGTTCATTGCGAAACG |  |
| *ctgfa* | F: CTACGGCTCCCCAAGTAACC  R: TCCACTGCGGTACACCATTC |  |
| *cyr61* | F: ACAAGCTGCAACCTACCACT  R: AGAGTATTCATTCTACTCACACTCA |  |
| *actb2* | F: GCTGTTTTCCCCTCCATTGTT  R: TCCCATGCCAACCATCACT |  |
| *gmfg* | F: TGTGAGGTGGACGATGGCCT  R: CTGCATGTCGATCTTCATCAGG |  |
| *gmfb* | F: GTGTGAGGTGGACGAGGACCT  R: CATGCTCCTCCTCCAGGATTACC |  |
| *gmfg*  (full length) | F: cttgttctttttgcaggatccATGTCGAGCGCTCTGGTTGT  R:gaattcgaatcgatgggatccTTAGCGGAAAAAGGAGAGTTTCTG |  |

**Supplementary Table4 Western blot antibodies used in this study.**

| Name | Dilution rate | Catalog Number | Brand |
| --- | --- | --- | --- |
| anti-Runx1 Ab | 1:1000 | A2055 | ABclonal |
| anti-Cmyb Ab | 1:500 | MCA1793 | AbD Serotec |
| anti-Gmfg Ab | 1:1000 | 13625-1-AP | Proteintech |
| anti-Gmfb Ab | 1:1000 | 10690-1-AP | Proteintech |
| anti-YAP | 1:500 | sc-101199 | Santa Cruz |
| anti-pYAP (phospho S127) | 1:5000 | ab76252 | Abcam |
| anti-CTGF Ab | 1:1000 | 23936-1-AP | Proteintech |
| anti-KLF2 Ab | 1:1000 | ab236507 | Abcam |
| anti-β-actin Ab | 1:1000 | EM21002 | Huabio |
| anti-GAPDH Ab | 1:2000 | 5174S | Cell Signaling Technology |
| anti-laminB1 Ab | 1:1000 | A01090 | Abbkine Scientific |

**Supplementary Table5 chemicals used in this study.**

| Name | Targeted signaling | type | Stock/work concentration | refence |
| --- | --- | --- | --- | --- |
| dorsomorphin | BMP signaling | inhibitor | 10mM/10µM | [4] |
| cyclopamine | Hedgehog signaling | inhibitor | 100mM/100µM | [5] |
| forskolin | cAMP signaling | activator | 1mM/1µM | [6] |
| DAPT | Notch signaling | inhibitor | 100mM/100µM | [7] |

**Supplementary Table6 shRNAs used in this study.**

| Name | Sequence (from 5’ to 3’) |
| --- | --- |
| ctl-sh | CCGGGGTTCTCCGAACGTGTCACGTCTCGAGACGTGACACGTTCGGAGAACCTTTTTG |
| *gmfg*-sh1 | CCGGGTTCGTGGTTTACAGCTACAACTCGAGTTGTAGCTGTAAACCACGAACTTTTTT |
| *gmfg*-sh2 | CCGGTGGCCGAGTGTCCTACCCTTTCTCGAGAAAGGGTAGGACACTCGGCCATTTTTT |
| *gmfg*-sh3 | CCGGGAGTGTCCTACCCTTTGTGTTCTCGAGAACACAAAGGGTAGGACACTCTTTTTT |
| *gmfg*-sh4 | CCGGATGATGTATGCAGGGAGTAAACTCGAGTTTACTCCCTGCATACATCATTTTTTT |

**REFERENCE**

1 Zuo P, Fu Z, Tao T, Ye F, Chen L, Wang X *et al*. The expression of glia maturation factors and the effect of glia maturation factor-gamma on angiogenic sprouting in zebrafish. *Exp Cell Res* 2013; 319: 707-717.

2 Becker JR, Deo RC, Werdich AA, Panakova D, Coy S, MacRae CA. Human cardiomyopathy mutations induce myocyte hyperplasia and activate hypertrophic pathways during cardiogenesis in zebrafish. *Dis Model Mech* 2011; 4: 400-410.

3 Espin-Palazon R, Stachura DL, Campbell CA, Garcia-Moreno D, Del Cid N, Kim AD *et al*. Proinflammatory signaling regulates hematopoietic stem cell emergence. *Cell* 2014; 159: 1070-1085.

4 Zhang C, Lv J, He Q, Wang S, Gao Y, Meng A *et al*. Inhibition of endothelial ERK signalling by Smad1/5 is essential for haematopoietic stem cell emergence. *Nat Commun* 2014; 5: 3431.

5 Gering M, Patient R. Hedgehog signaling is required for adult blood stem cell formation in zebrafish embryos. *Dev Cell* 2005; 8: 389-400.

6 Jing L, Tamplin OJ, Chen MJ, Deng Q, Patterson S, Kim PG *et al*. Adenosine signaling promotes hematopoietic stem and progenitor cell emergence. *J Exp Med* 2015; 212: 649-663.

7 He Q, Zhang C, Wang L, Zhang P, Ma D, Lv J et al. Inflammatory signaling regulates hematopoietic stem and progenitor cell emergence in vertebrates. Blood 2015; 125: 1098-110
