# Supplementary material for: Glia maturation factor-γ is required for initiation and maintenance of hematopoietic stem and progenitor cells
Source: Stem Cell Res Ther. 2023 Apr 30;14:117. doi: 10.1186/s13287-023-03328-1 (PMC10150485; doi:10.1186/s13287-023-03328-1)

Additional File 2

Glia maturation factor-γ is required for initiation and maintenance of hematopoietic stem and progenitor cells

By

Honghu Li[1,2,3,4,*], Qian Luo[1,2,3,4,*], Shuyang Cai[1,2,3,4,*], Ruxiu Tie[1,2,3,4],Ye Meng[1,2,3,4], Wei Shan[1,2,3,4], Yulin Xu[1,2,3,4], Xiangjun Zeng[1,2,3,4], Pengxu Qian[1.2.3.4.5.6✉], He Huang[1,2,3,4.5✉]

*These authors contributed equally to this work: Honghu Li, Qian Luo, Shuyang Cai.

Original, unprocessed scans of blots:

Figure S1 Runx1

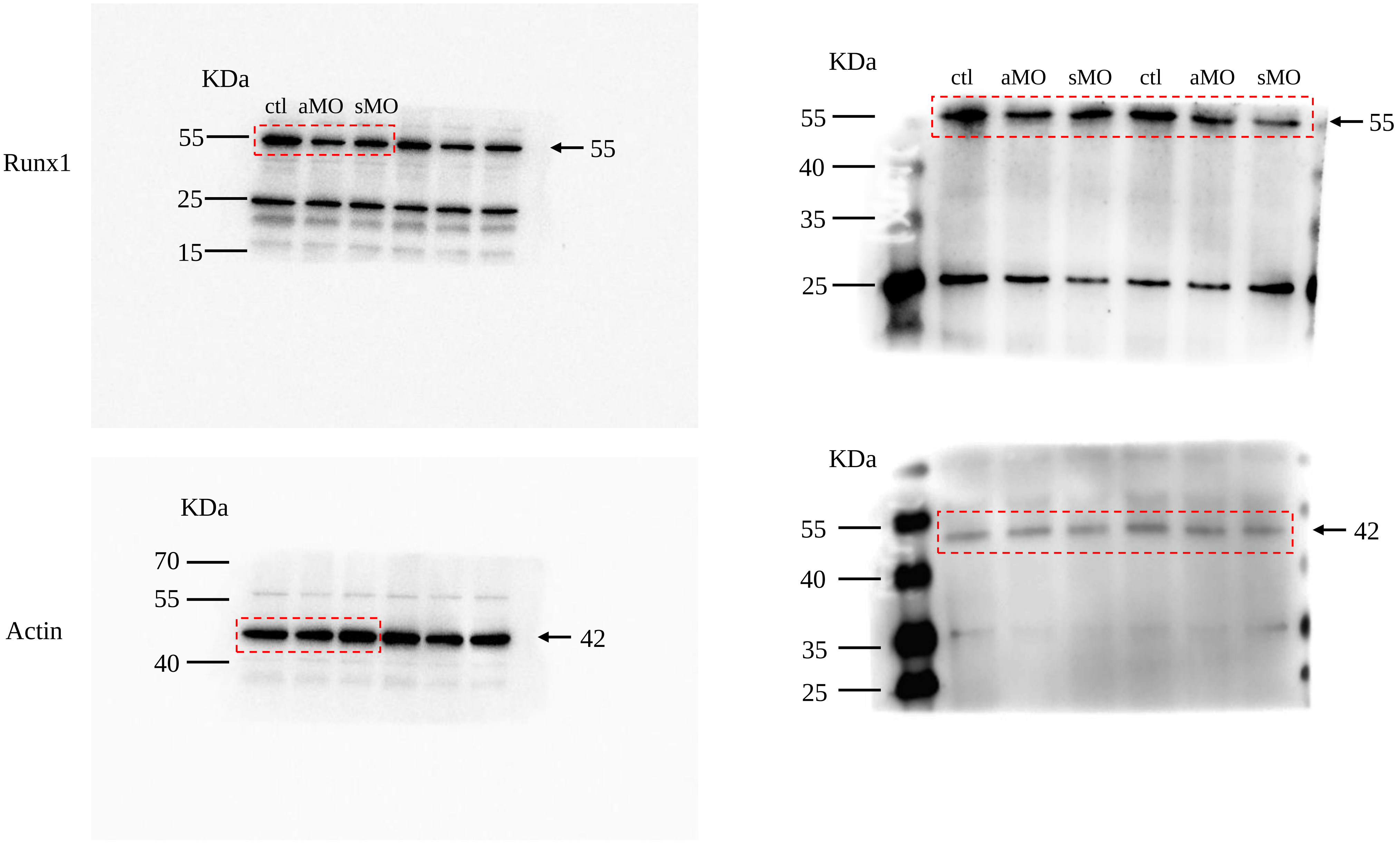

Figure S2 Cmyb

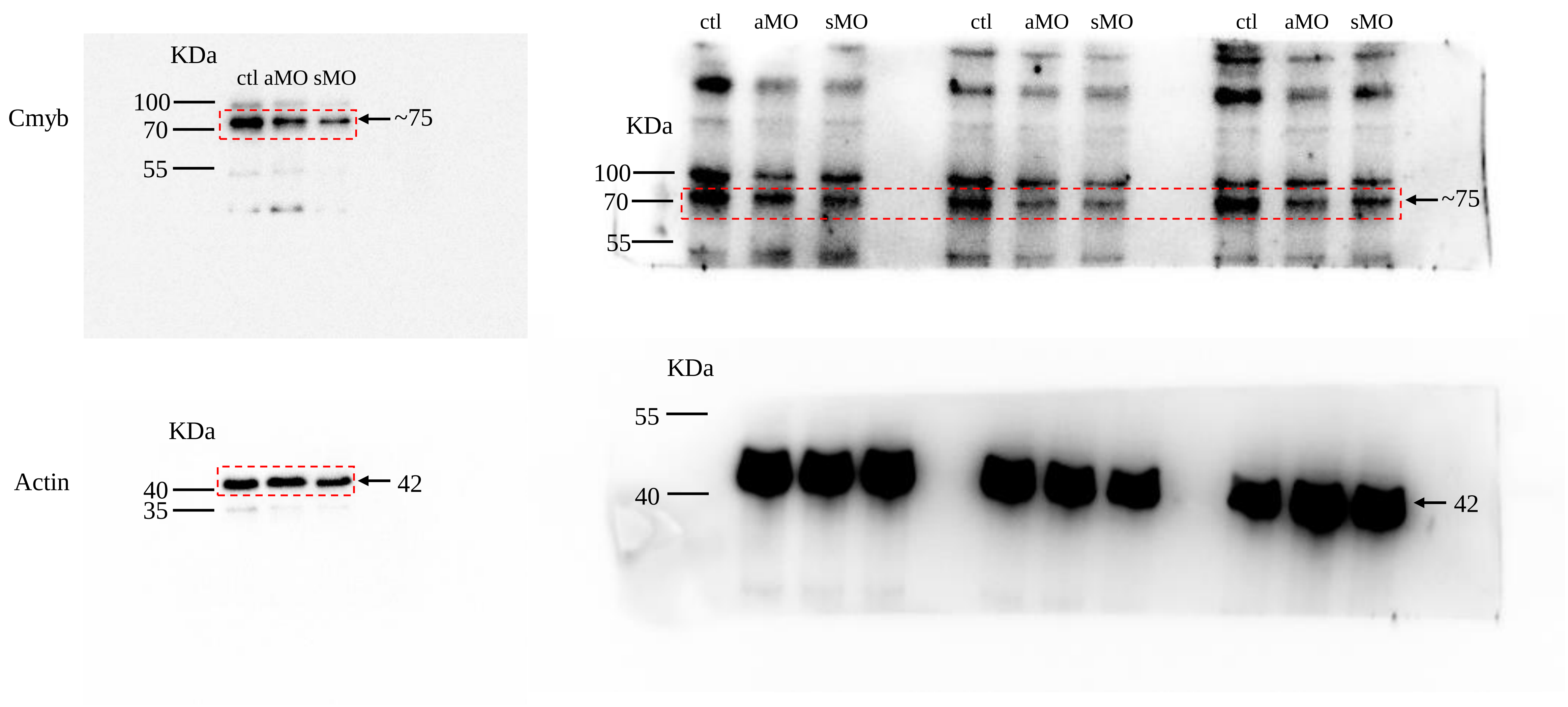

Figure S3 Gmfg

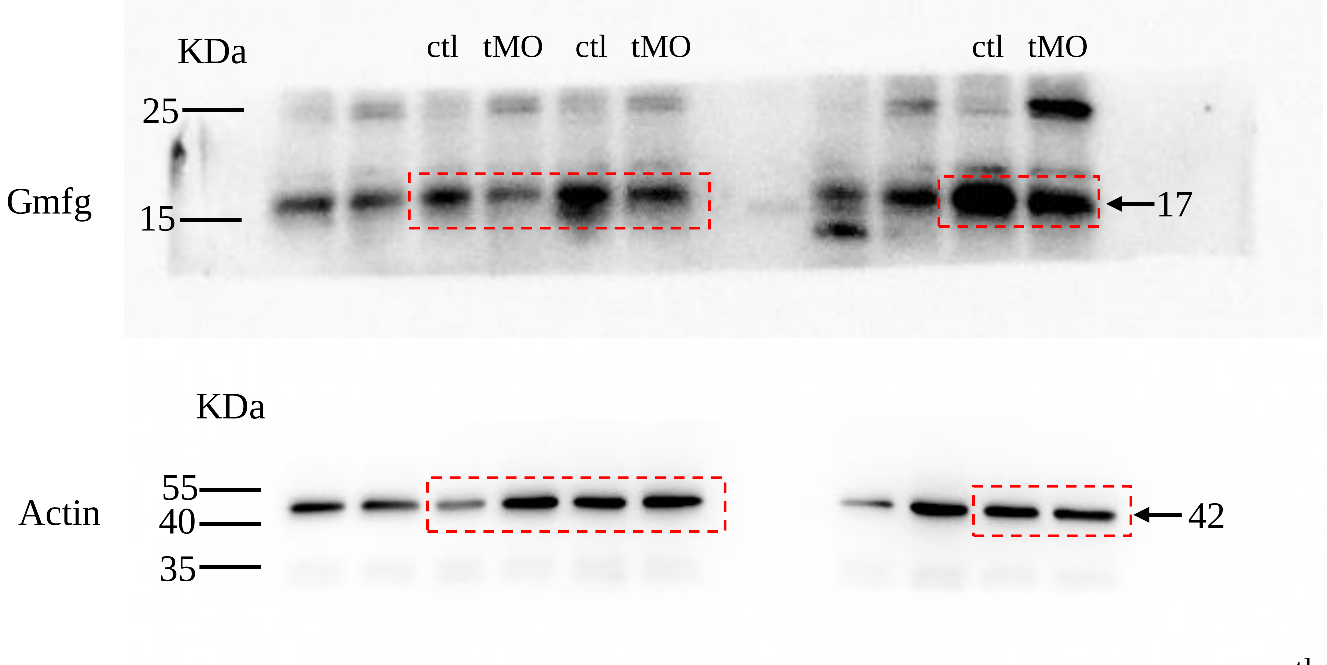

Figure S4 Klf2a

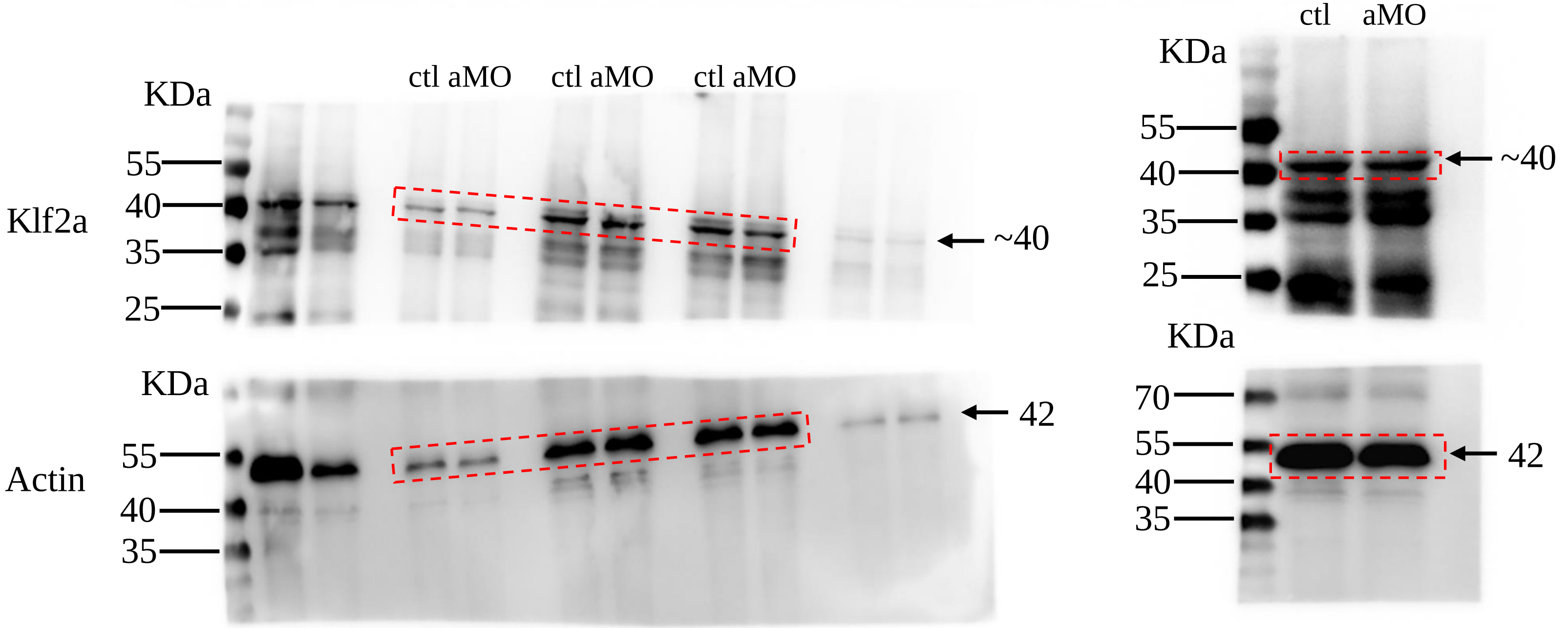

Figure S5 Yap

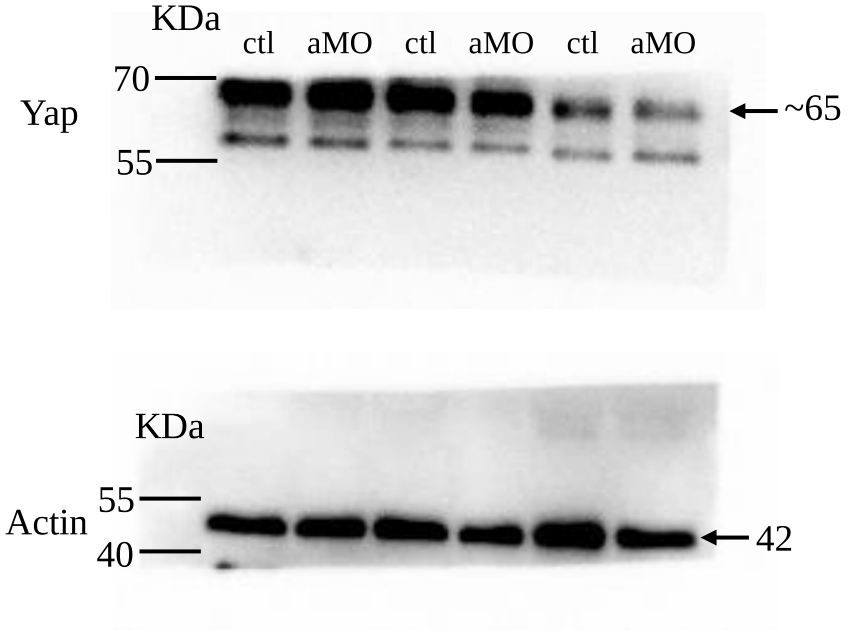

Figure S6 Ctgf

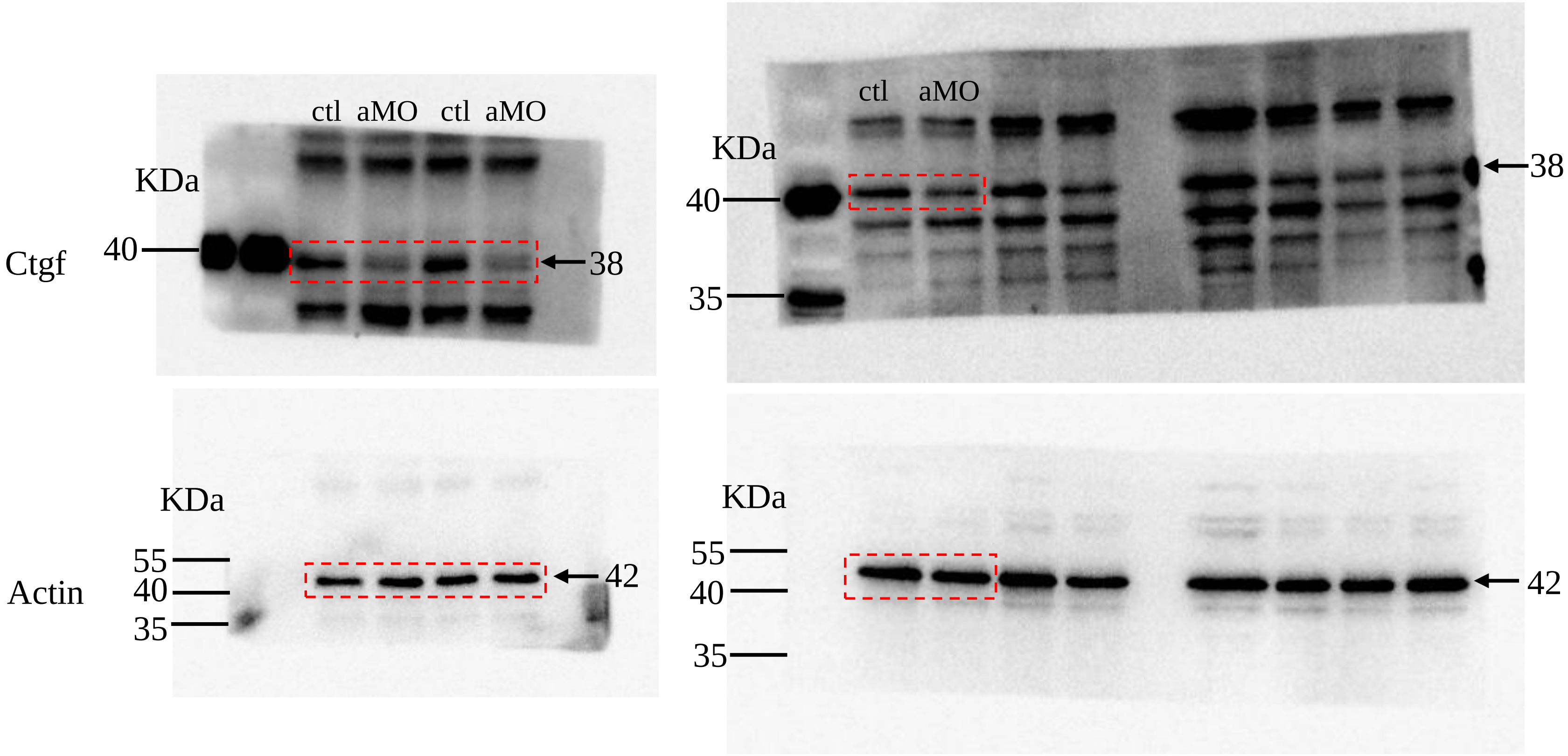

Figure S7 p-Yap(S127)

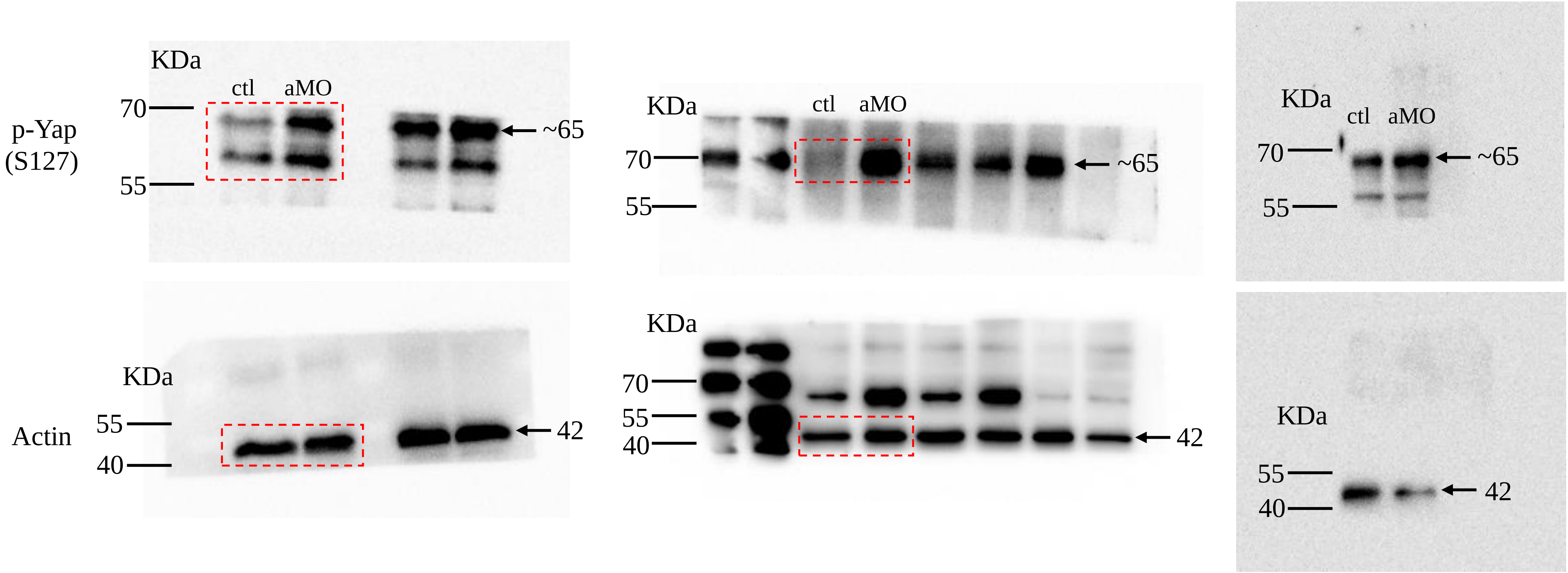

Figure S8 GMFG

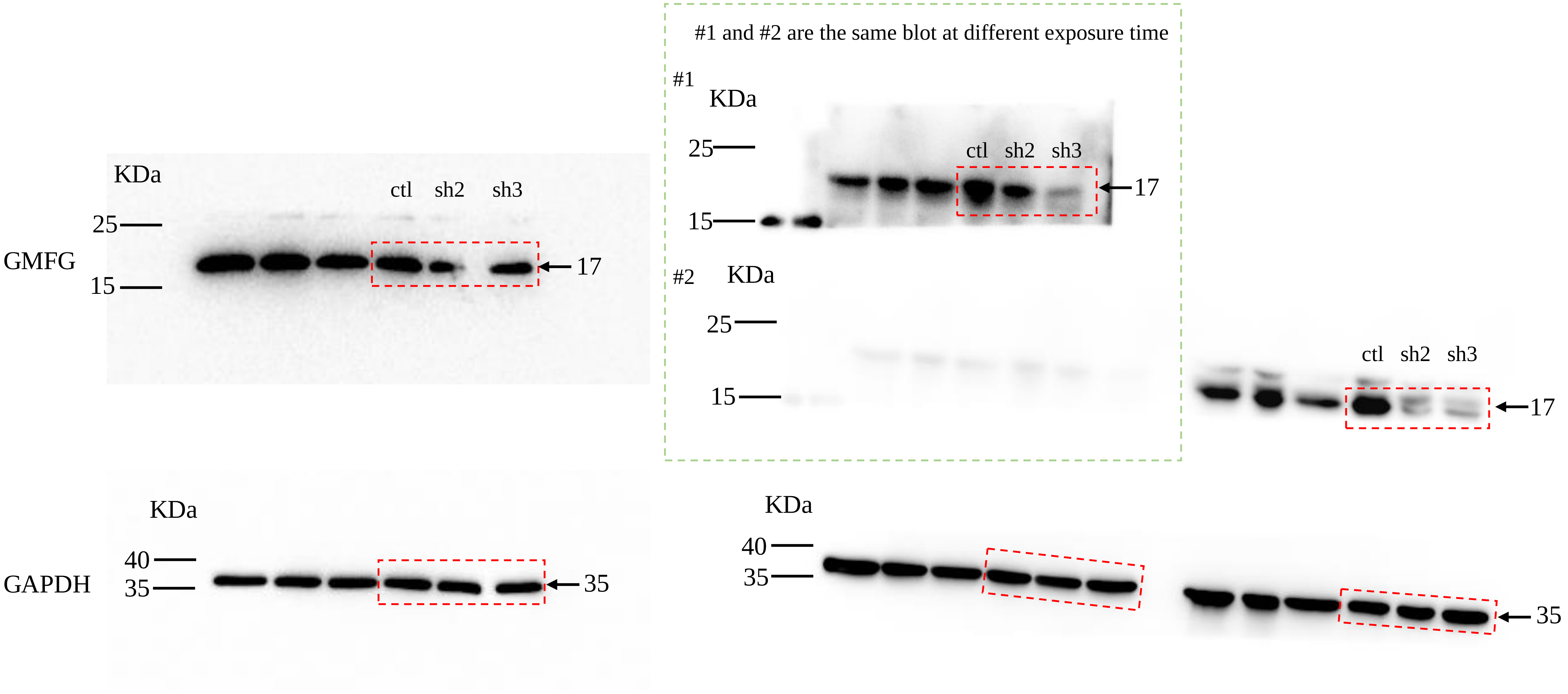

Figure S9 YAP

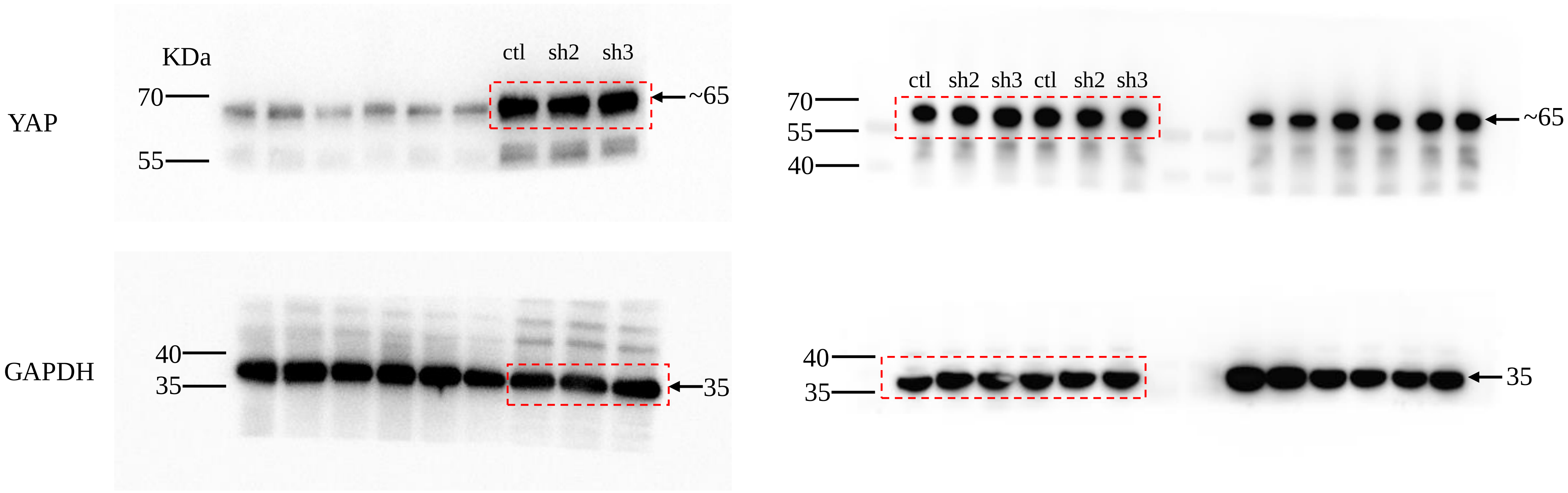

Figure S10 CTGF

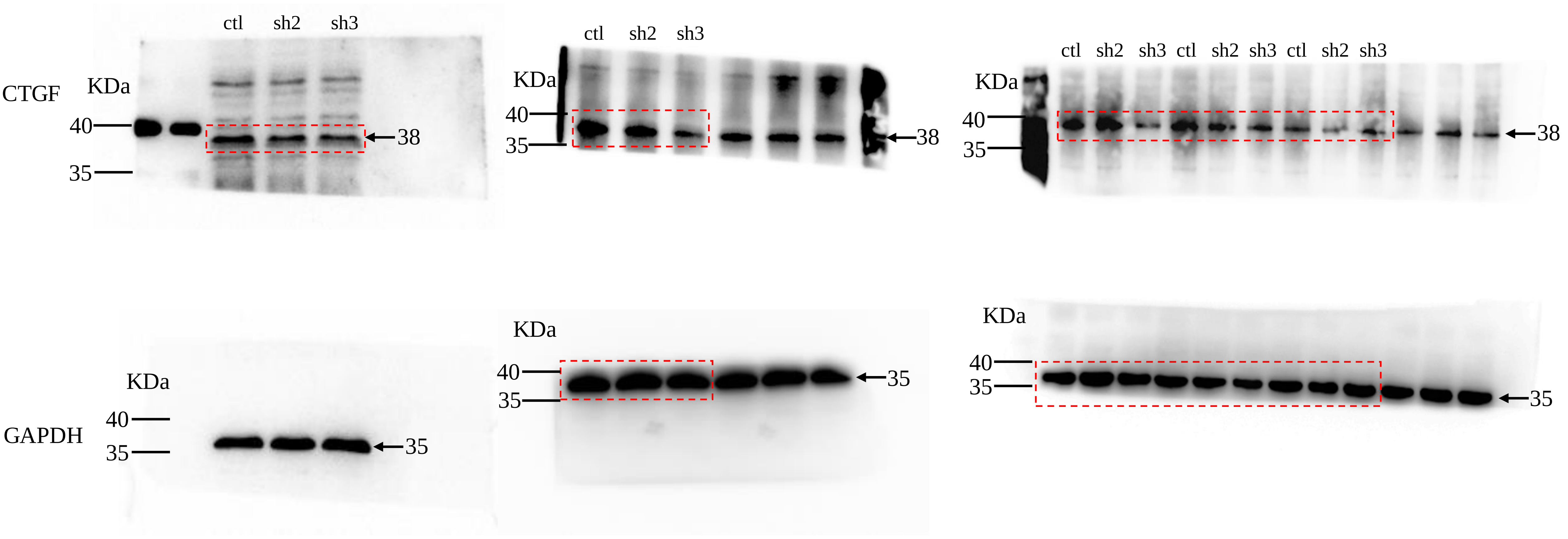

Figure S11 nuclei YAP

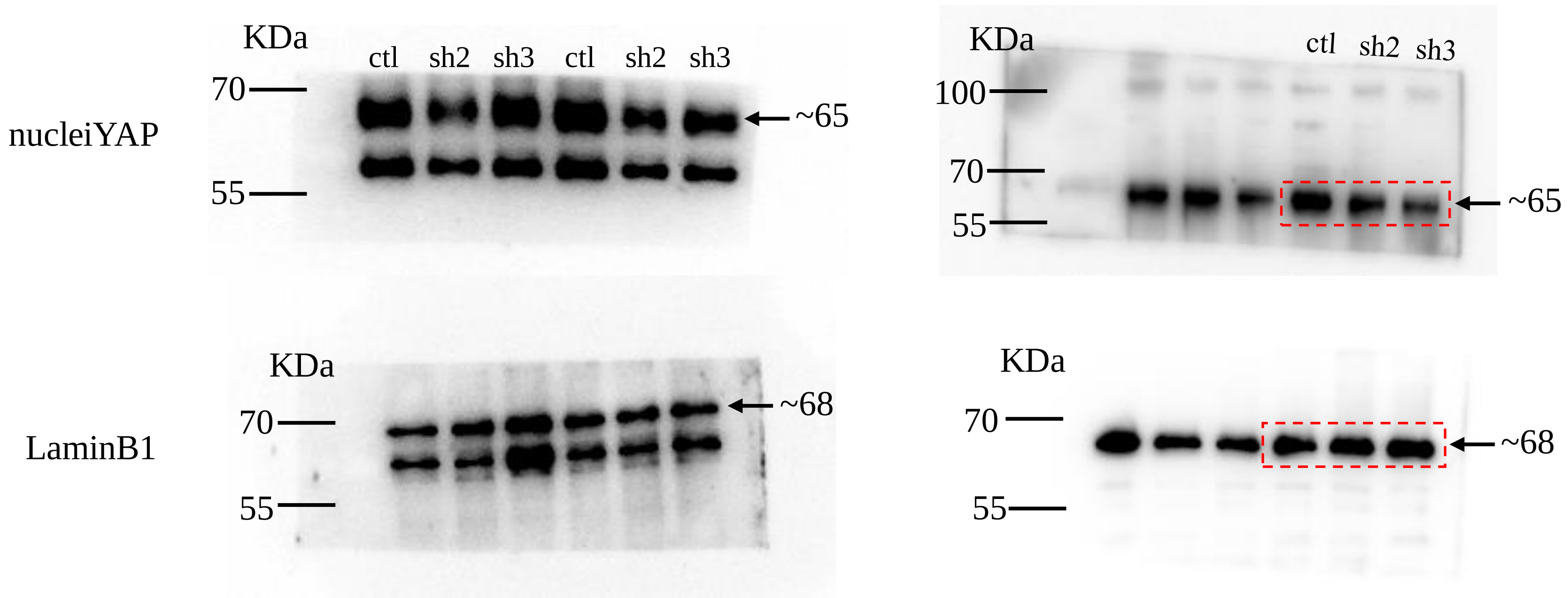

Figure S12 cyto YAP

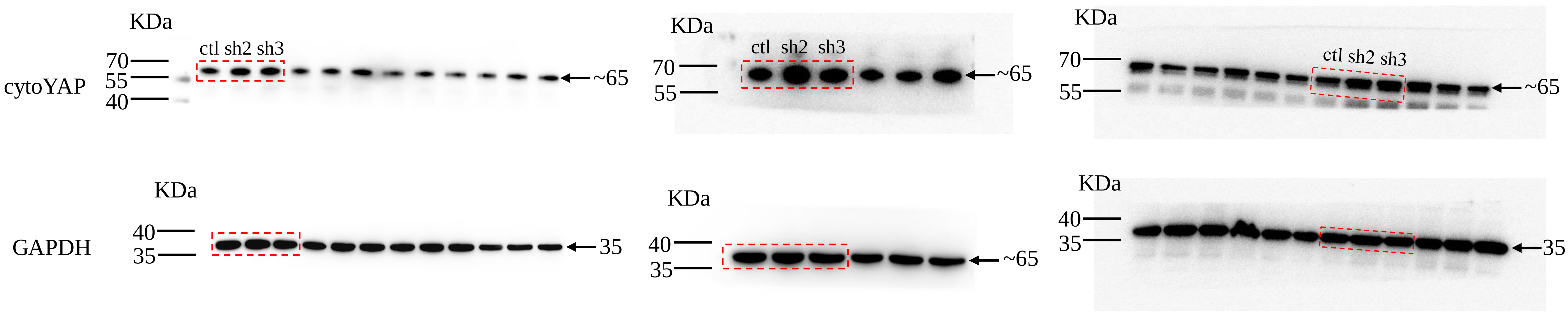

Figure S13 Gmfg-36 hpf

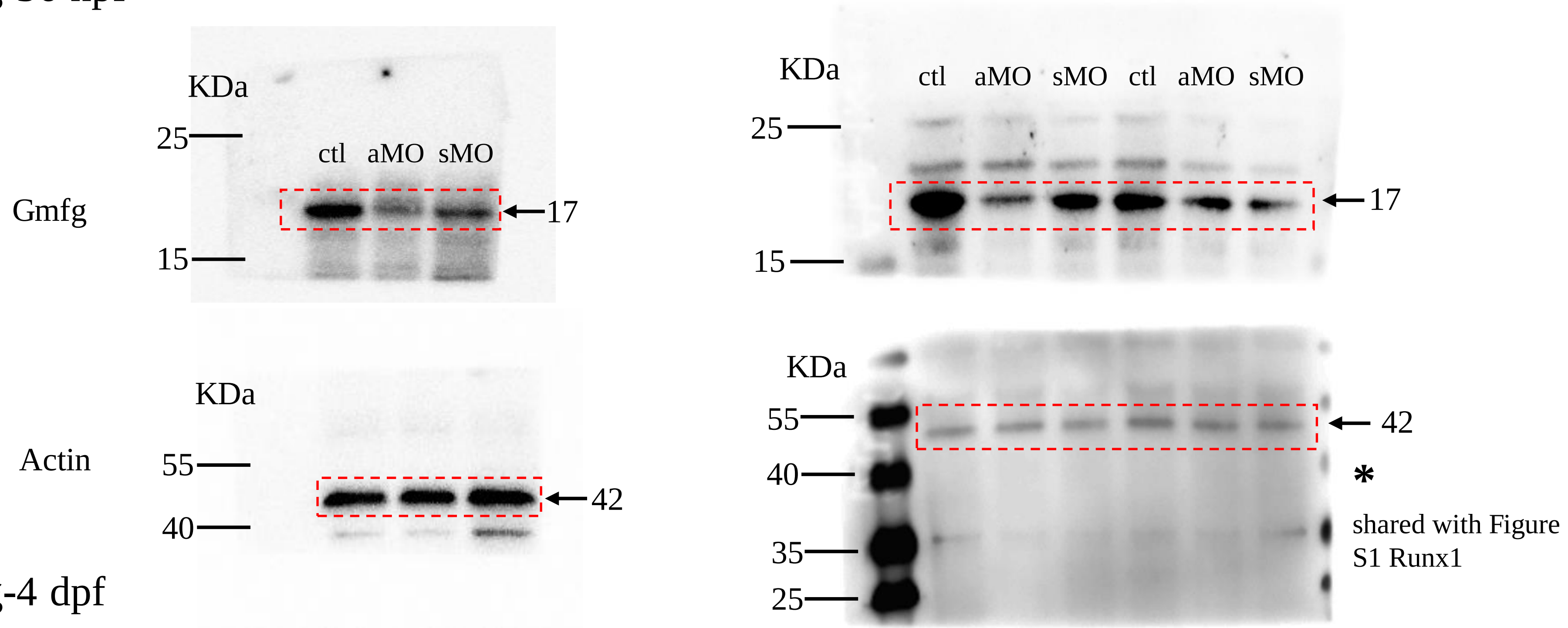

Figure S14 Gmfg-4 dpf

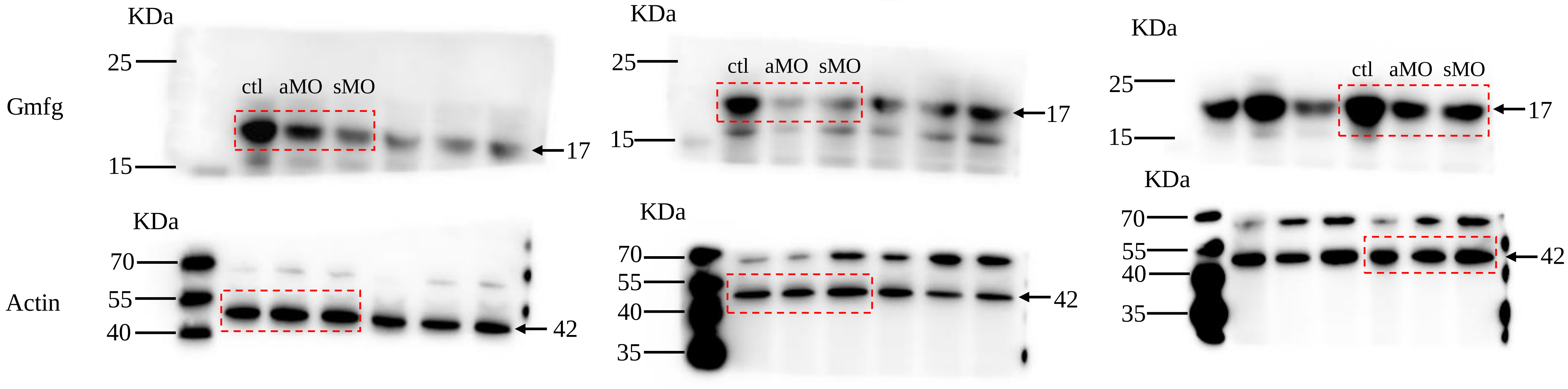

Figure S15 Gmfb

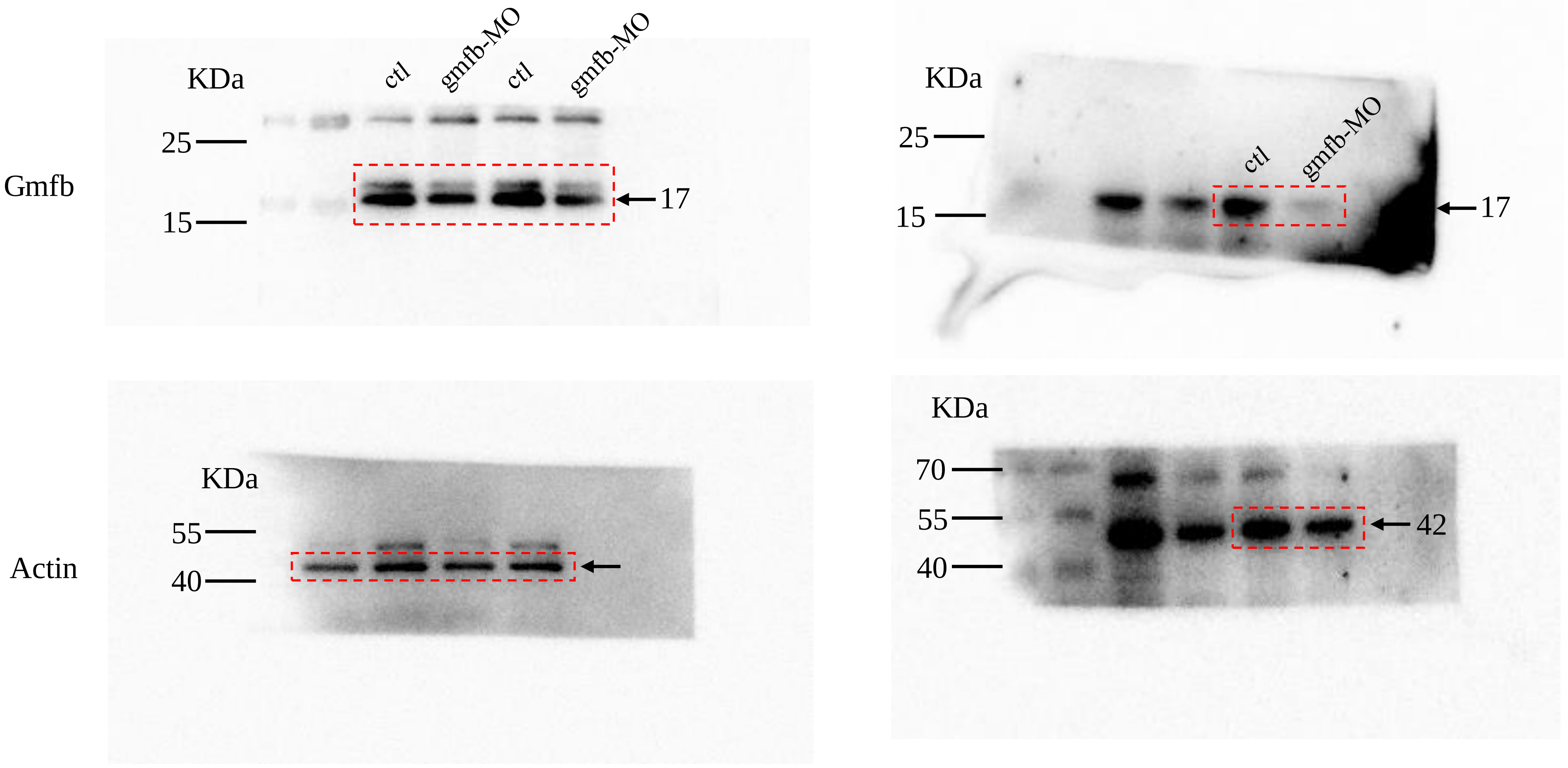

Figure S16 Gmfg

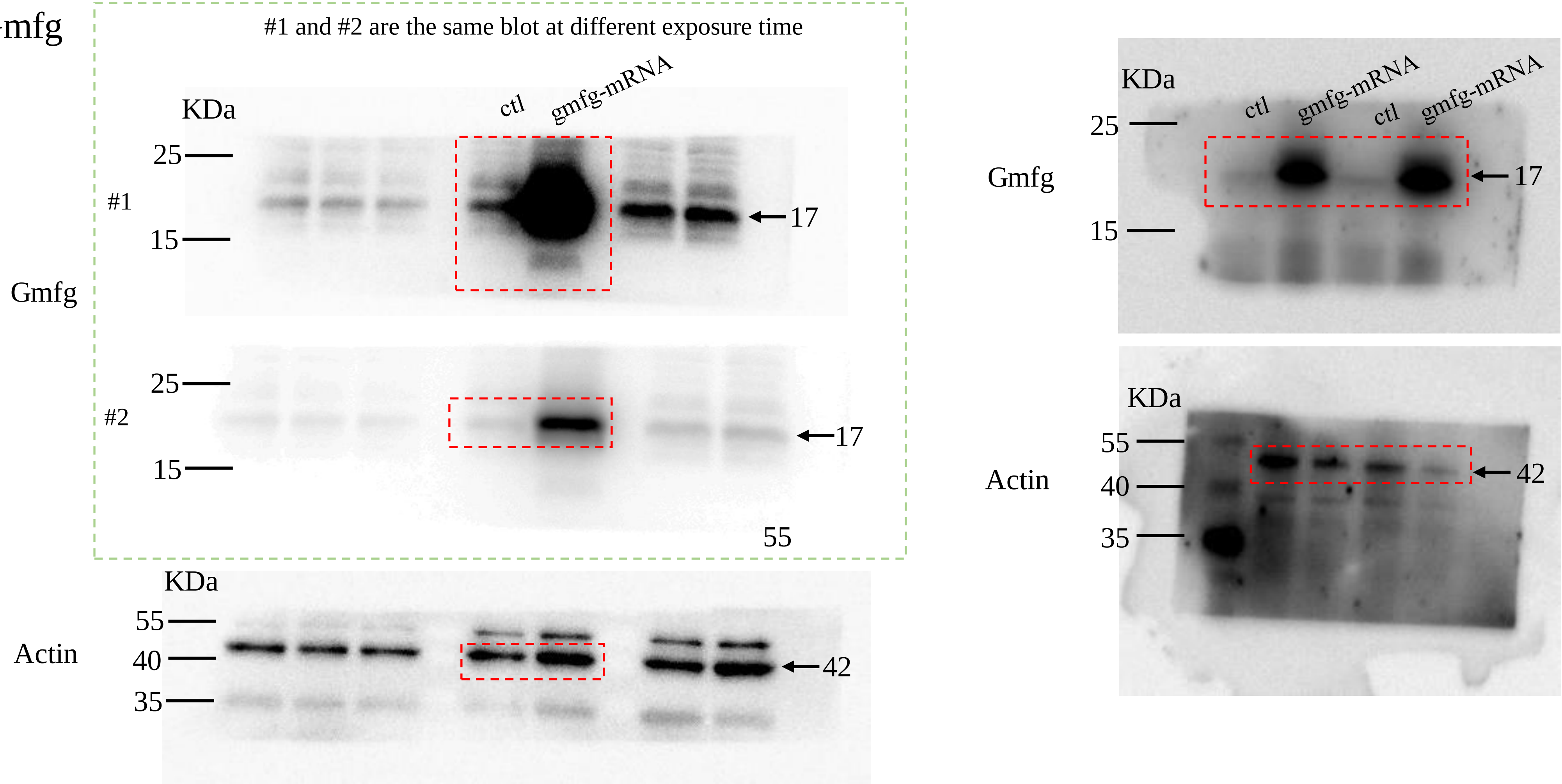

Figure S17 Actin/Gapdh

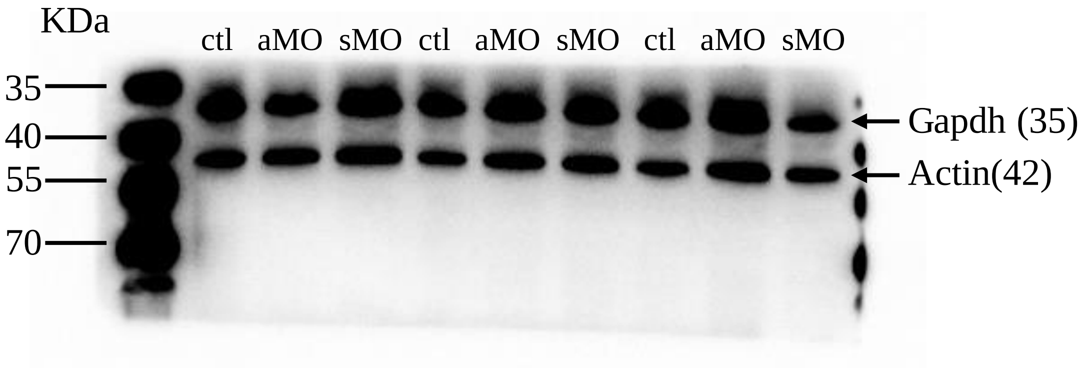

Supplement: Supplementary file 2 — Additional file 2. Corresponding original, unprocessed scans of blots. The original, unprocessed images of blots shown in Figure 2b, Figure 5b, Figure 6c, Figure 6e, Figure 6f, Supplementary Figure 1a, Supplementary Figure 1b, Supplementary Figure 2b, and Supplementary Figure 3 are listed. ctl denotes control group, aMO denotes gmfg-atgMO group, sMO denotes gmfg-spMO group, tMO denotes tnnt2a-MO group, sh2 denotes gmfg-sh2 group and sh3 denotes gmfg-sh3 group. [file 13287_2023_3328_MOESM2_ESM.pdf]
